# Secreted peptidases contribute to virulence of fish pathogen *Flavobacterium columnare*

**DOI:** 10.3389/fcimb.2023.1093393

**Published:** 2023-02-03

**Authors:** Nicole C. Thunes, Haitham H. Mohammed, Jason P. Evenhuis, Ryan S. Lipscomb, David Pérez-Pascual, Rebecca J. Stevick, Clayton Birkett, Rachel A. Conrad, Jean-Marc Ghigo, Mark J. McBride

**Affiliations:** ^1^ Department of Biological Sciences, University of Wisconsin-Milwaukee, Milwaukee, WI, United States; ^2^ Department of Rangeland, Wildlife and Fisheries Management, Texas A&M University, College Station, TX, United States; ^3^ National Center for Cool and Cold Water Aquaculture, Agricultural Research Service, United States Department of Agriculture, Kearneysville, WV, United States; ^4^ Institut Pasteur, Université de Paris-Cité, Centre National de la Recherche Scientifique (CNRS) Unité Mixte de Recherche (UMR) 6047, Genetics of Biofilms Laboratory, Paris, France

**Keywords:** *flavobacterium*, fish pathogen, gene deletion, type IX secretion system (T9SS), protease

## Abstract

*Flavobacterium columnare* causes columnaris disease in freshwater fish in both natural and aquaculture settings. This disease is often lethal, especially when fish population density is high, and control options such as vaccines are limited. The type IX secretion system (T9SS) is required for *F. columnare* virulence, but secreted virulence factors have not been fully identified. Many T9SS-secreted proteins are predicted peptidases, and peptidases are common virulence factors of other pathogens. T9SS-deficient mutants, such as Δ*gldN* and Δ*porV*, exhibit strong defects in secreted proteolytic activity. The *F. columnare* genome has many peptidase-encoding genes that may be involved in nutrient acquisition and/or virulence. Mutants lacking individual peptidase-encoding genes, or lacking up to ten peptidase-encoding genes, were constructed and examined for extracellular proteolytic activity, for growth defects, and for virulence in zebrafish and rainbow trout. Most of the mutants retained virulence, but a mutant lacking 10 peptidases, and a mutant lacking the single peptidase TspA exhibited decreased virulence in rainbow trout fry, suggesting that peptidases contribute to *F. columnare* virulence.

## Introduction

*Flavobacterium columnare* is a Gram-negative bacterium belonging to the phylum *Bacteroidota* that causes columnaris disease in freshwater fish (Declercq et al., 2013). This is of particular concern in aquaculture, where high population density of fish leads to greater chances of infection and mortality (Suomalainen et al., 2005; Wagner et al., 2006; Pulkkinen et al., 2010). *F. columnare* colonizes the gills, skin, and fins, resulting in skin lesions, pigment loss, fin erosion, and respiratory distress (Decostere et al., 1999; Wagner et al., 2006; Pulkkinen et al., 2010; Declercq et al., 2013). Virulence mechanisms of *F. columnare* are poorly understood, and adequate control and prevention measures are lacking.

The type IX secretion system (T9SS) is required for *F. columnare* virulence (Li et al., 2017; Thunes et al., 2022). The T9SS secretes soluble and cell-surface associated proteins (Sato et al., 2010; Shrivastava et al., 2013). Proteins secreted by the *F. columnare* T9SS are potential virulence factors, and most have not been studied. These proteins typically have a signal peptide (SP) at the N-terminus and a conserved C-terminal domain (CTD) at the opposite end that is required for secretion (McBride, 2019). The function of the CTD was first identified during studies of gingipain protease secretion by another member of the *Bacteroidota*, *Porphyromonas gingivalis* (Seers et al., 2006). Secreted proteins use the Sec system to cross the cytoplasmic membrane, and then use the T9SS to transit the outer membrane. Three types of CTDs are found on different T9SS-secreted proteins: type A (TIGR04183), type B (TIGR04131), and type C (cl41395) (Kulkarni et al., 2017; Lu et al., 2020; Thunes et al., 2022).

Some proteins that lack recognizable CTDs may also require the T9SS to leave the cell. This was suggested by liquid chromatography-tandem mass spectrometry (LC-MS/MS) experiments that found such proteins in the cell-free spent media of wild-type cultures but not in those from a T9SS-deficient mutant (Li et al., 2017; Thunes et al., 2022). These nonCTD proteins may be recognized by the T9SS by a different mechanism, or their secretion may be only indirectly affected by loss of the T9SS. For example, the T9SS may secrete a cell surface component of another secretion system that handles the nonCTD proteins. Alternatively, the unnatural accumulation of proteins with T9SS-CTDs in the periplasm of a T9SS-defective mutant could interfere with another secretion system or could otherwise impact the cell surface and alter secretion. Regardless of the cause, these extracellular ‘nonCTD’ proteins that require the T9SS for their apparent secretion are also possible virulence factors.

Of the previously identified secreted *F. columnare* proteins in cell-free spent media, thirty-five were identified as predicted peptidases, and eleven of these had obvious T9SS CTDs (Thunes et al., 2022). Such secreted peptidases with T9SS-CTDs are referred to in this paper as ‘CTD-peptidases’. Peptidases (also referred to as proteases) are secreted by bacteria, fungi, and other organisms (Clarke et al., 2016). They are often used for nutrient acquisition and may also contribute to virulence (Mckerrow et al., 1993; Klemba and Goldberg, 2002; Kolar et al., 2013; Ruiz-Perez and Nataro, 2014; Yang et al., 2015; Zhang et al., 2015). *P. gingivalis* secretes its major virulence factors, gingipain proteases, *via* its T9SS (Vincent et al., 2017; Benedyk et al., 2019). *F. columnare* secreted peptidases may contribute to the skin lesions and necrosis that are characteristic signs of columnaris disease (Newton et al., 1997; Dumpala et al., 2010). Targeting peptidases as treatment or preventative strategies has been effective for some pathogens (Klemba and Goldberg, 2002; Clarke et al., 2016), providing a rationale to study *F. columnare* secreted peptidases.

The fish pathogen *Flavobacterium psychrophilum* also secretes many peptidases that were proposed as potential virulence factors (Duchaud et al., 2007). Mutation of several peptidase-encoding genes reduced proteolytic activities but had no effect on virulence in rainbow trout (Pérez-Pascual et al., 2011; Gómez et al., 2015). For both *F. psychrophilum* and *F. columnare* the large number of predicted secreted peptidases makes it difficult to determine which, if any, contribute to virulence. It also suggests the possibility that partial redundancy may occur between some of the peptidases.

Recently, *F. columnare* strain MS-FC-4 was shown to be more amenable to genetic manipulation than other strains examined (Thunes et al., 2022). This allowed efficient construction of strains with multiple genes deleted (Thunes et al., 2022; Conrad et al., 2022a; Conrad et al., 2022b). Here we took advantage of the relative ease of genetic manipulation and constructed a series of gene deletion mutants to explore the roles of 20 peptidases in *F. columnare* virulence in fish.

## Results

### Identification of apparent T9SS-secreted peptidases and deletion of the genes encoding these

Cell-free spent culture fluid of wild-type *F. columnare* strain MS-FC-4 was previously analyzed for proteins that were secreted by wild-type cells, but were secreted poorly, if at all, by the T9SS-deficient mutant Δ*gldN* (Thunes et al., 2022). Thirty-five secreted proteins predicted to be peptidases were identified in that study. We reexamined the data from that study and identified three additional predicted peptidases (C6N29_07780, C6N29_08680, and C6N29_08590) (**Table S1**). Twelve of the 38 secreted peptidases had CTDs predicted to target them for secretion by the T9SS. We refer to these as ‘CTD-peptidases’, and the corresponding peptidase-encoding genes as ‘CTD-peptidase genes’. Ten CTD-peptidase genes were deleted, generating mutants lacking these genes (**Tables 1**, **S1**, **S2**). Deletions were constructed sequentially, resulting in ‘chain mutants’ lacking up to ten CTD-peptidase genes. Two of the CTD-peptidase genes (*porU* and C6N29_09900) were not deleted in this study. PorU functions in secretion, where it cleaves T9SS CTDs (Glew et al., 2012). Because T9SS mutants have already been characterized for virulence defects (Li et al., 2017; Thunes et al., 2022), we did not focus on this peptidase in this study, and thus did not delete *porU*. We attempted, but failed, to delete C6N29_09900 from chain mutant FCB167, which lacks 10 other CTD-peptidase genes. We did not delete five other CTD-peptidase genes (C6N29_02760, C6N29_05620, C6N29_08010, C6N29_12610, C6N29_12705), for which the encoded proteins were not detected in cell-free culture fluid (Thunes et al., 2022).

**Table 1:** Peptidase mutants used in this study^a^.

Strain name	Descriptive name	Locus tag deleted	Reference
CTD-peptidase chain mutants			
FCB20	Δ1-CTD-P	Deletion of C6N29_05800 from WT	(Thunes et al., 2022)
FCB98	Δ2-CTD-P	Deletion of C6N29_05315 in FCB20	This study
FCB105	Δ3-CTD-P	Deletion of C6N29_07780 in FCB98	This study
FCB109	Δ4-CTD-P	Deletion of C6N29_10335 in FCB105	This study
FCB134	Δ5-CTD-P	Deletion of C6N29_10910 in FCB109	This study
FCB139	Δ6-CTD-P	Deletion of C6N29_04605 in FCB134	This study
FCB142	Δ7-CTD-P	Deletion of C6N29_03390 in FCB139	This study
FCB145	Δ8-CTD-P	Deletion of C6N29_09865 in FCB142	This study
FCB155	Δ9-CTD-P	Deletion of C6N29_08145 in FCB145	This study
FCB167	Δ10-CTD-P	Deletion of C6N29_13645 in FCB155	This study
nonCTD-peptidase chain mutants			
FCB54	Δ2-nonCTD-P	Deletion of C6N29_11545, C6N29_11550 and C6N29_05800 from WT	(Thunes et al., 2022)
FCB100	Δ3-nonCTD-P	Deletion of C6N29_00585 in FCB54	This study
FCB101	Δ4-nonCTD-P	Deletion of C6N29_08590 in FCB100	This study
FCB102	Δ5-nonCTD-P	Deletion of C6N29_12020 in FCB101	This study
FCB103	Δ6-nonCTD-P	Deletion of C6N29_14605 in FCB102	This study
FCB107	Δ7-nonCTD-P	Deletion of C6N29_03570 in FCB103	This study
FCB117	Δ8-nonCTD-P	Deletion of C6N29_12115 in FCB107	This study
FCB137	Δ9-nonCTD-P	Deletion of C6N29_06855 in FCB117	This study
Additional deletion mutants missing single peptidase genes			
FCB135	Δ*tspA*	Deletion of C6N29_08680 (Δ*tspA*) encoding predicted tail-specific nonCTD-protease from WT	This study
FCB147	ΔC6N29_12115	Deletion of C6N29_12115 encoding predicted nonCTD-peptidase from WT	This study
FCB159	ΔC6N29_06855	Deletion of C6N29_06855 encoding predicted nonCTD-peptidase from WT	This study
FCB222	ΔC6N29_08145	Deletion of C6N29_08145 encoding predicted CTD-peptidase from WT	This study
FCB230	ΔC6N29_13645	Deletion of C6N29_13645 encoding predicted CTD-peptidase from WT	This study

a For details of mutant construction see Methods and **Table S2**.

A second chain deletion mutant was constructed lacking genes encoding peptidases that appeared to require the T9SS for secretion but that lacked obvious T9SS CTDs. We refer to these secreted peptidases as ‘nonCTD-peptidases’ (**Table 1**). Genes were prioritized primarily based on number of spectral counts of gene products in cell-free culture fluid from wild-type cells compared to those of the T9SS-deficient Δ*gldN* mutant (**Table S1**). Both chain mutants started with deletion of the CTD-peptidase gene, ΔC6N29_05800. A mutant (FCB54) lacking this gene and also lacking two nonCTD-peptidase genes (ΔC6N29_11545 and ΔC6N29_11550) was previously shown to exhibit decreased proteolytic activity (Thunes et al., 2022). FCB54 was chosen as the background to delete additional nonCTD-peptidase genes, resulting in the ‘nonCTD-peptidase chain’ mutants. Sequential deletions resulted in chain mutants lacking up to nine nonCTD-peptidase genes, each of which also lacked the CTD-peptidase gene ΔC6N29_05800 (**Table 1**).

Conserved domains associated with peptidases were identified in the proteins encoded by the CTD-peptidase genes and nonCTD-peptidase genes that were deleted in this study (**Table 2**). The proteins were diverse, belonging to different peptidase families. Fourteen were predicted to be metallopeptidases. Sixteen of the peptidases are similar in sequence to peptidases of the related fish pathogen, *F. psychrophilum* (**Table 2**).

**Table 2:** Predicted domains and activities of *F. columnare* MS-FC-4 peptidases examined in this study.

Locus tag; gene name	Protein ID	AA^a^	Conserved domains^b^	Description/predicted function^c^	Related *F. psychrophilum* proteins^d^
CTD-peptidases^e^					
C6N29_03390	PTD13559.1	682	LasB super family (cl34569) GluZincin super family (cl14813)	Probable peptidase; Zinc metalloprotease; Thermolysin-like proteinases	
C6N29_04605	PTD13770.1	1076	ZnMc super family (cl00064) myxo_dep_M36 super family (cl45606) FN3 (cd00063)	Probable peptidase Zinc metalloprotease	FP0231 (Fpp1), 42% ID/912 AA (Secades et al., 2001; Pérez-Pascual et al., 2011)
C6N29_05315	PTD13902.1	2135	Peptidases_S8_S53 super family (cl0459) denti_PrtP super family (cl41341) CUB (smart00042) Laminin_G_3 (pfam13385)	Possible peptidase	
C6N29_05800	PTD13986.1	902	FTP (pfam07504) M4_TLP (cd09597)	Probable metallopeptidase, M4 family	
C6N29_07780	PTD14344.1	1107	Sortilin-Vps10 super family (cl25791) PA_subtilisin_1 (cd04818)	possible subtilisin-like protease	
C6N29_08145	PTD14409.1	910	T9SSA_dep_M36 super family (cl45607)	Probable metallopeptidase, M36 family	FP0280, 45% ID/952 AA; FP0281, 47% ID/950 AA (Duchaud et al., 2007)
C6N29_09865	PTD14714.1	422	ZnMc super family (cl00064)	Probable peptidase, Zinc metalloprotease	FP1619, 30% ID/314 AA (Duchaud et al., 2007)
C6N29_10335	PTD14805.1	943	ZnMc_pappalysin_like (cd04275) choice_anch_J super family (cl45621)	Probable peptidase, Zinc metalloprotease	FP0232 (Fpp2), 54% ID/952 AA (Secades et al., 2003; Pérez-Pascual et al., 2011)
C6N29_10910	PTD14907.1	438	ZnMc super family (cl00064)	Probable peptidase, Zinc metalloprotease	FP1777, 41% ID/379 AA (Duchaud et al., 2007)
C6N29_13645	PTD15387.1	995	FTP (pfam07504) M4_TLP (cd09597)	Probable metallopeptidase, M4 family	FP0086, 38% ID/401AA (Duchaud et al., 2007)
nonCTD peptidases					
C6N29_00585	PTD16151.1	705	PreP super family (cl34286)	S9A family serine peptidase	FP0870, 28% ID/683 AA
C6N29_03570	PTD13591.1	719	Peptidase_S46 (pfam10459)	S46 family peptidase	FP0382, 70% ID/696 AA
C6N29_06855	PTD14169.1	462	Zinc_peptidase_like super family (cl14876)	subfamily M20F dipeptidase, metallopeptidase	FP0150, 85% ID/462 AA
C6N29_08590	PTD14484.1	274	Peptidase_M48_M56 super family (cl28898)	subfamily M48C metallopeptidase	FP0300, 67% ID/263 AA
C6N29_08680; *tspA*	PTD14500.1	723	PRK11186 super family (cl36004)	tail-specific protease (carboxy terminal-processing protease)	FP2333, 69% ID/709 AA
C6N29_11545	PTD15009.1	301	Zn_peptidase super family (cl19825)	Metalloprotease	FP0506, 61% ID/294 AA (Rochat et al., 2019)
C6N29_11550	PTD15010.1	300	Zn_peptidase super family (cl19825)	Metalloprotease	FP0506, 56% ID/296 aa (Rochat et al., 2019)
C6N29_12020	PTD15097.1	317	ZnMc_pappalysin_like (cd04275)	Zinc metalloprotease	FP1619, 63% ID/330 AA (Duchaud et al., 2007)
C6N29_12115	PTD15115.1	317	ZnMc_pappalysin_like (cd04275)	Zinc metalloprotease	FP1619, 63% ID/330 AA (Duchaud et al., 2007)
C6N29_14605	PTD15568.1	722	DPPIV_N super family (cl37636) DAP2 super family (cl34287)	S9 family peptidase	FP1112, 43% ID over 702 AA

a Length of full-length proteins in amino acids before removal of signal peptide or CTD.

b Conserved domains other than signal peptide and CTDs are shown, and were assigned by NCBI and by the Joint Genome Institute Integrated Microbial Genomes & Microbiomes (IMG/M version 6.0 [https://img.jgi.doe.gov/m]) (Chen et al., 2021). pfam, smart, cl, or cd, numbers are indicated.

c Description/predicted function based on conserved domains, and or as assigned by NCBI or

d Related proteins from F. psychrophilum as indicated by the locus tags and by the papers cited.

e Each of the CTD-peptidases has a predicted Type A T9SS-CTD as recognized by their relationship to protein domain family TIGR04183.

### Deletion of multiple CTD-peptidase genes reduced extracellular proteolytic activity

**Figure 1:**
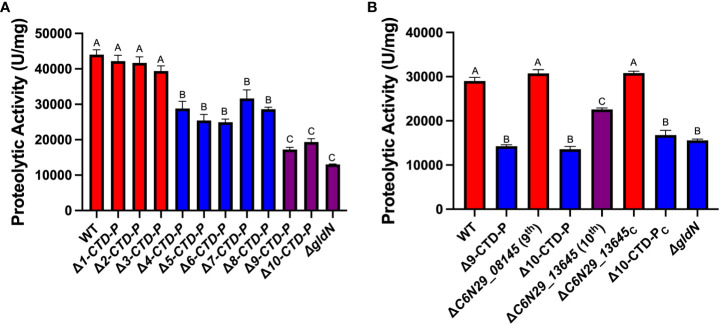
Effect of deletion of genes encoding peptidases with T9SS CTDs on extracellular proteolytic activities. **(A)** Strains examined were wild-type *F. columnare* (WT); FCB20 (Δ1-CTD-P); FCB98 (Δ2-CTD-P); FCB105 (Δ3-CTD-P); FCB109 (Δ4-CTD-P); FCB134 (Δ5-CTD-P); FCB139 (Δ6-CTD-P); FCB142 (Δ7-CTD-P); FCB145 (Δ8-CTD-P); FCB155 (Δ9-CTD-P); FCB167 (Δ10-CTD-P); Δ*gldN*. **(B)** Strains examined were wild-type *F. columnare* (WT); FCB155 (Δ9-CTD-P); FCB222 (ΔC6N29_08145); FCB167 (Δ10-CTD-P); FCB230 (ΔC6N29_13645); ΔC6N29_13645 mutant with wild-type C6N29_13645 restored to the chromosome (ΔC6N29_13645_C_); Δ10-CTD-P mutant with wild-type C6N29_13645 restored to the chromosome (Δ10-CTD-P_C_); Δ*gldN*. Statistics correspond to one-way ANOVA with Tukey post-test comparing all conditions to wild type and each other. Same letter: nonsignificant.

Wild-type and CTD-peptidase gene deletion mutants were examined for extracellular proteolytic activity (**Figure 1A**). The T9SS-deficient Δ*gldN* mutant, which is deficient in secretion of peptidases targeted to the T9SS (Thunes et al., 2022), was also included in this analysis. In most cases, the sequential deletion of CTD-peptidase genes resulted in a partial decline in extracellular proteolytic activity. The largest changes occurred upon deletion of the fourth (C6N29_10335) and ninth (C6N29_08145) of the CTD-peptidase genes that were targeted. The mutants lacking 9 or 10 peptidase-encoding genes showed severe defects in extracellular proteolytic activity compared to wild type. These defects were similar to those of the T9SS-defective Δ*gldN* mutant.

We deleted the 9^th^ (C6N29_08145) and 10^th^ (C6N29_13645) CTD-peptidase genes from wild-type cells to determine if either of these contributed a large amount of extracellular proteolytic activity by itself. Deletion of C6N29_08145 from wild-type cells did not cause a significant reduction in proteolysis, whereas deletion of C6N29_13645 caused a partial reduction of proteolytic activity (**Figure 1B**). A wild-type copy of C6N29_13645 (the 10^th^ CTD-peptidase) was recombined into its native site on the chromosome of the single mutant (ΔC6N29_13645) and of the Δ10-CTD-P chain mutant to determine if secreted proteolytic activity was restored. This restored proteolysis to the single mutant (ΔC6N29_13645) but not to the Δ10-CTD-P mutant. The defects in the Δ9-CTD-P and Δ10-CTD-P mutants appear to be caused by the cumulative effects of the deletion of multiple peptidase-encoding genes rather than primarily by the loss of either C6N29_08145 or C6N29_13645.

### Deletion of the nonCTD-peptidase gene C6N29_12115 reduced extracellular proteolytic activity

**Figure 2:**
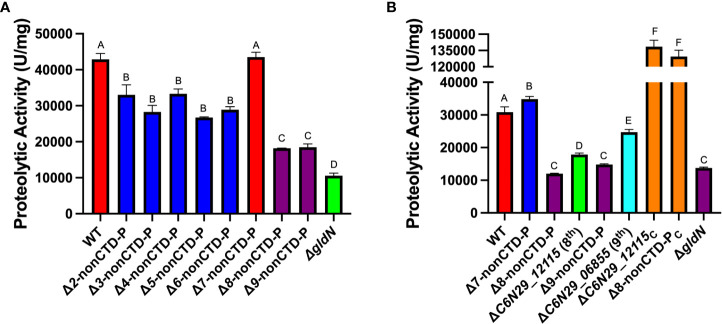
Effect of deletion of genes encoding peptidases that lack T9SS CTDs on extracellular proteolytic activities. **(A)** Strains examined were wild-type *F. columnare* (WT); FCB54 (Δ2-nonCTD-P); FCB100 (Δ3-nonCTD-P); FCB101 (Δ4-nonCTD-P); FCB102 (Δ5-nonCTD-P); FCB103 (Δ6-nonCTD-P); FCB107 (Δ7-nonCTD-P); FCB117 (Δ8-nonCTD-P); FCB137 (Δ9-nonCTD-P); Δ*gldN*. **(B)** Strains examined were WT; FCB107 (Δ7-nonCTD-P); FCB117 (Δ8-nonCTD-P); FCB147 (ΔC6N29_12115); FCB137 (Δ9-nonCTD-P); FCB159 (ΔC6N29_06855); ΔC6N29_12115 complemented with plasmid pHM23 (ΔC6N29_12115_C_); Δ8-nonCTD-P complemented with plasmid pHM23 (Δ8-nonCTD-P_C_); Δ*gldN*. Statistics correspond to one-way ANOVA with Tukey post-test comparing all conditions to wild type and each other. Same letter and color: nonsignificant.

NonCTD-peptidase gene deletion chain mutants were also examined for extracellular proteolytic activity (**Figure 2**). These mutants had deletions spanning genes that lacked identifiable T9SS-associated CTDs, but each also lacked one CTD-peptidase gene, ΔC6N29_05800. The parent strain FCB54 (which we refer to here as Δ2-nonCTD-P), lacking a CTD-peptidase gene and two nonCTD-peptidase genes, exhibited less extracellular proteolytic activity than did wild type, as was previously reported (Thunes et al., 2022). Mutants lacking up to 6-nonCTD-peptidase genes displayed levels of extracellular proteolytic activity similar to the parent strain, FCB54. Deletion of the 7^th^ nonCTD-peptidase gene C6N29_14605 restored proteolytic activity to near wild type levels. In contrast, deletion of the eighth nonCTD-peptidase gene C6N29_12115, resulted in decreased proteolytic activity.

Single deletion mutants lacking the 8^th^ (C6N29_12115) and 9^th^ (C6N29_06855) nonCTD-peptidase genes were constructed, as were complemented strains. Deletion of the 8^th^ (C6N29_12115) or 9^th^ (C6N29_06855) nonCTD-peptidase genes from wild-type cells each caused a reduction in proteolysis compared to wild type. Complementation by introduction of multicopy plasmid pHM23 carrying C6N29_12115 into the single deletion mutant (ΔC6N29_12115) and into the Δ8-nonCTD-P mutant resulted in extracellular proteolytic activity in excess of that exhibited by wild-type cells (**Figure 2B**).

### Deletion of predicted tail-specific protease (ΔC6N29_08680) did not cause a decrease in proteolytic activity

C6N29_08680 is listed as a potential peptidase-encoding gene in the MEROPS peptidase database (https://www.ebi.ac.uk/merops/) (Rawlings et al., 2018). Unlike the other peptidase-encoding genes studied here it encodes a predicted tail-specific protease, and we thus refer to the gene as *tspA*. The encoded protein, TspA, lacks a T9SS CTD. In Gram-negative bacteria, tail-specific proteases, also referred to as carboxy-terminal processing proteases, are typically periplasmic (Hoge et al., 2011). As the names imply, they typically cleave specific C-terminal regions from target proteins. They are thought to impact the bacterium primarily by their effects on these bacterial target proteins. A *tspA* deletion mutant was constructed and screened for proteolytic activity. The deletion mutant exhibited extracellular proteolytic activity per mg bacterial cell protein that was similar to that of the wild type (**Figure S1**).

### Deletion of multiple peptidase-encoding genes did not reduce virulence of *F. columnare* in zebrafish

**Figure 3:**
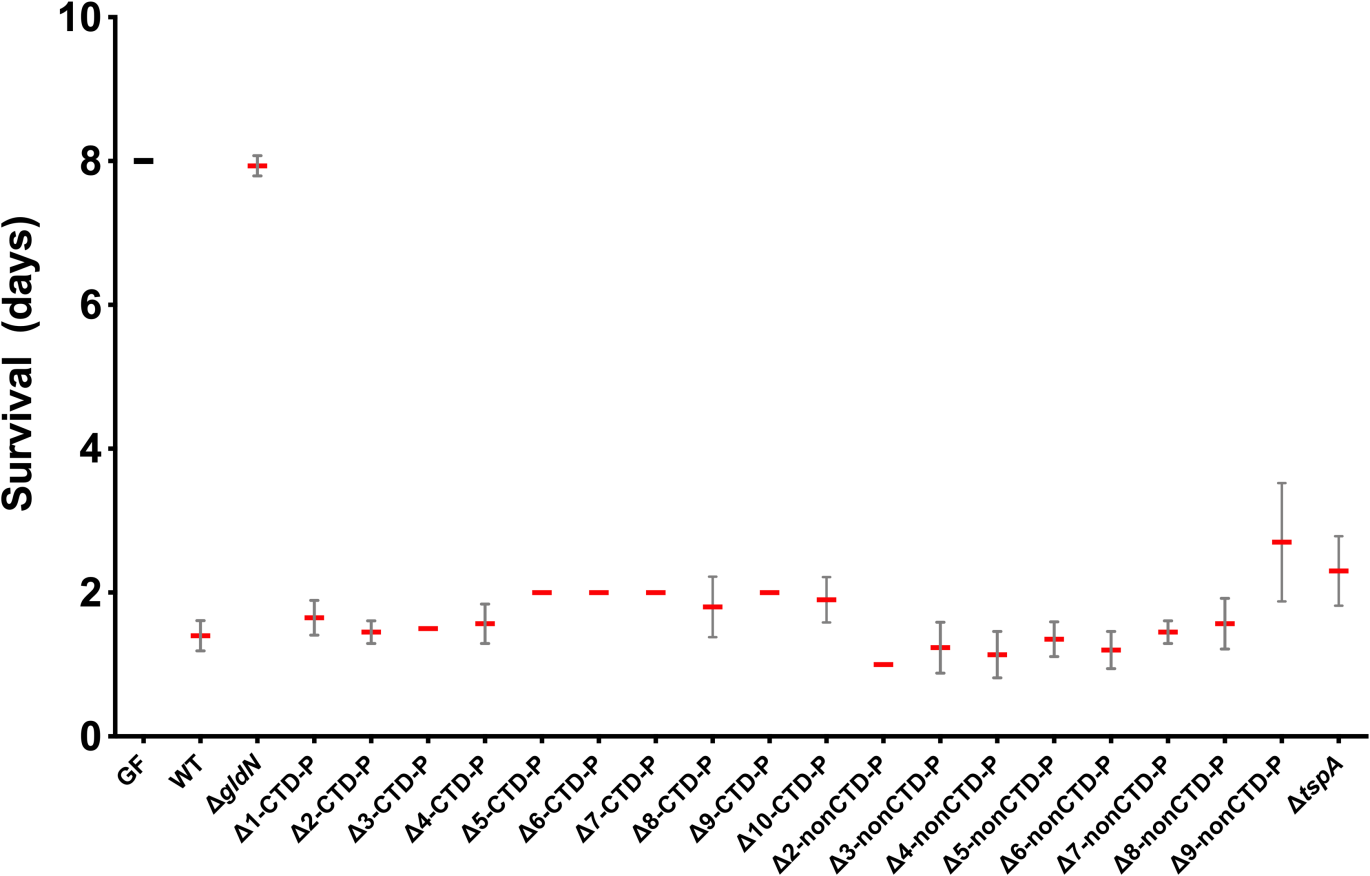
Virulence of *F. columnare* wild type and peptidase mutants toward germ-free (GF) zebrafish larvae. Fish were infected at 6 days post-fertilization by immersion at 10^4^ colony-forming-units (CFU)/mL. Zero days post-infection (dpi) corresponds to the day of infection. Mean survival is represented by a thick horizontal bar with error bars for standard deviation. “GF” (germ-free) indicates noninfected larvae. Length of survival is shown for larvae exposed to the strains indicated. The number of days of survival for fish challenged with any of the peptidase gene deletion mutant strains were not significantly different from those of the wild type. The survival for fish challenged with the *ΔgldN* mutant was not significantly different from the noninfected GF control.

Wild-type *F. columnare* and the peptidase mutants were examined for virulence against germ-free zebrafish larvae in a challenge experiment (**Figure 3**). No significant differences in virulence were seen for any of the mutants when compared to the wild type. In contrast, the T9SS-deficient Δ*gldN* mutant was avirulent, as previously reported (Thunes et al., 2022). A challenge experiment was also conducted for mutants lacking nine and ten CTD-peptidase genes (Δ9-CTD-P and Δ10-CTD-P, respectively) and a mutant lacking one CTD-peptidase gene and nine nonCTD-peptidase genes (Δ9-nonCTD-P) to examine virulence in adult zebrafish. No defects in virulence (as determined by monitoring percent survival after challenge) were observed in either case (**Figure S2**).

### Deletion of multiple peptidase-encoding genes caused decreased virulence of *F. columnare* in rainbow trout

**Figure 4:**
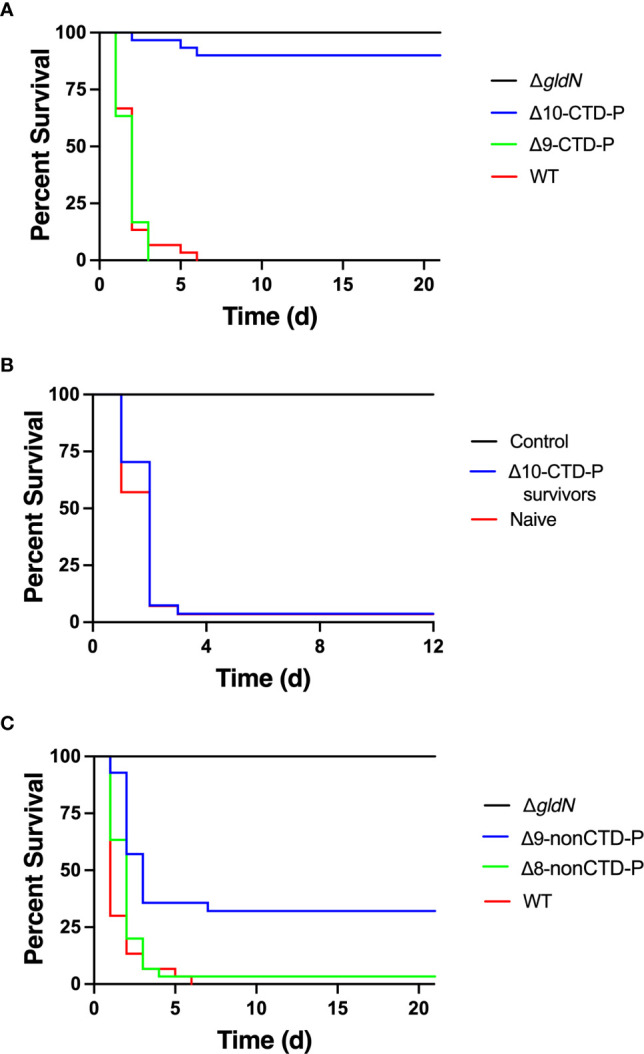
Effect of deletion of peptidase-encoding genes on virulence in juvenile rainbow trout. **(A)** Rainbow trout were exposed by immersion to wild-type and mutant strains and survival was monitored for 21 days. The final challenge concentrations were 3.4 × 10^7^ CFU/mL (WT), 6.6 × 10^5^ CFU/mL (Δ*gldN*), 2.6 × 10^7^ CFU/mL (Δ9-CTD-P), 1.3 × 10^7^ CFU/mL (Δ10-CTD-P). Significant differences in percent survival for fish challenged were observed between the WT and Δ10-CTD-P (*P*<0.0001). **(B)** Fish that survived exposure to Δ10-CTD-P (Δ10-CTD-P survivors) were examined for resistance to wild-type cells. Naïve fish and Δ10-CTD-P survivors were maintained for 28 d and then exposed to wild-type cells at 3.8 × 10^7^ CFU/ml. Survival was monitored for 12 d. Fish were exposed to TYES without bacteria as a control. **(C)** Rainbow trout were exposed by immersion to wild-type and mutant strains and survival was monitored. The final challenge concentrations were 3.4 × 10^7^ CFU/mL (WT), 6.6 × 10^5^ CFU/mL (Δ*gldN*), 3.6 × 10^7^ (Δ8-nonCTD-P), 2.7 × 10^7^ CFU/mL (Δ9-nonCTD-P). Significant differences in percent survival for fish challenged were observed between the WT and Δ9-nonCTD-P (*P*<0.0001).

**Figure 5:**
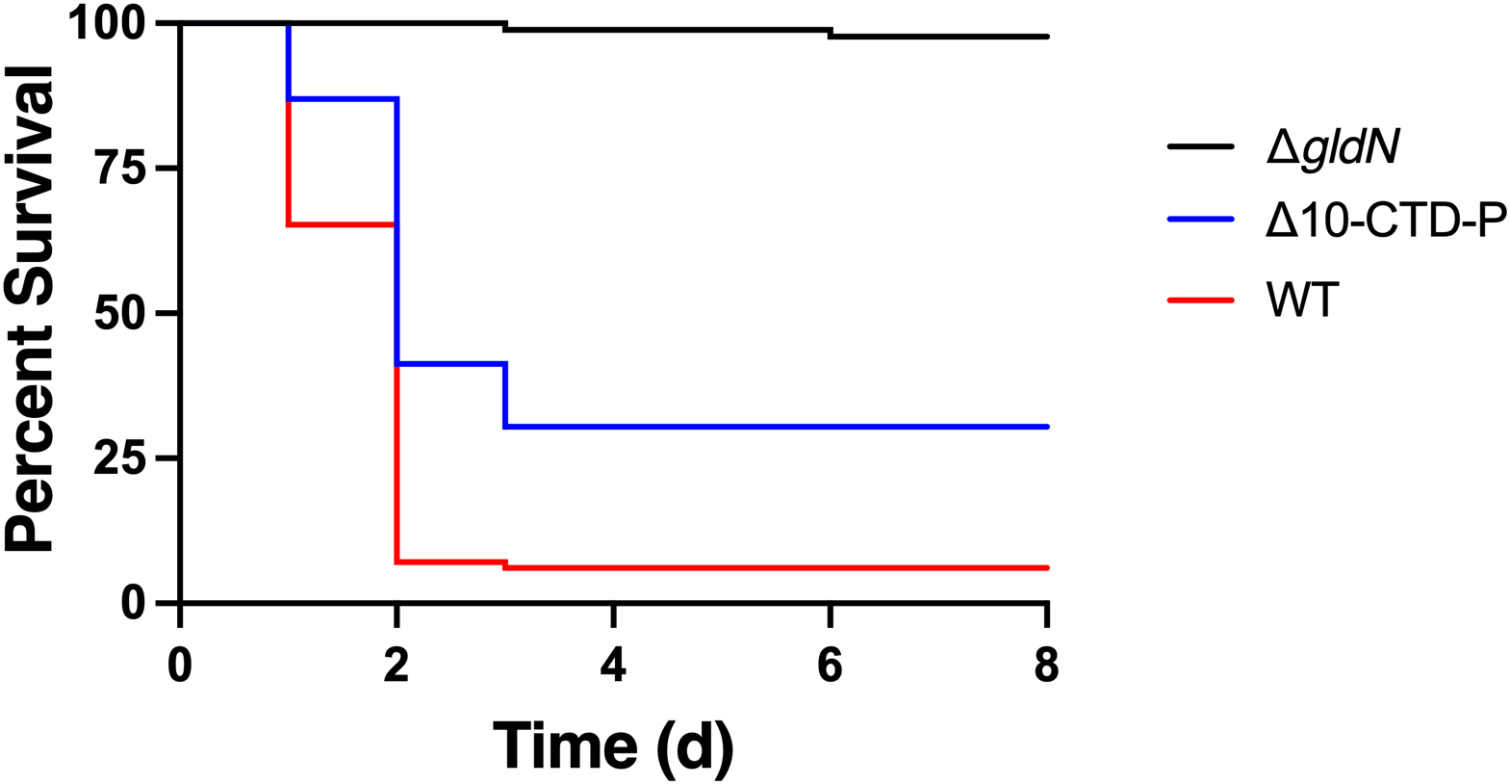
Effect of deletion of peptidase-encoding genes on virulence in rainbow trout alevin (sac-fry). Alevin were exposed by immersion to *F. columnare* strains and monitored for 8 d. The final challenge concentrations were 6.9 × 10^6^ CFU/mL (WT), 1.1 × 10^7^ CFU/mL (Δ*gldN*), and 8.0 × 10^6^ CFU/mL (Δ10-CTD-P). Significant differences in percent survival for fish challenged were observed between the WT and Δ10-CTD-P (*P*<0.0001).

Each CTD-peptidase chain mutant (**Table 1**) was tested for virulence in juvenile rainbow trout (also referred to as fry) (**Figure 4**). Only the Δ10-CTD-P chain mutant showed decreased virulence compared to wild type (**Figure 4A** and data not shown). Fish that survived exposure to Δ10-CTD-P were maintained for 28 days and then examined for sensitivity to challenge with the wild type. These fish were not protected against the later challenge (**Figure 4B**). Chain mutants in which nonCTD-peptidase-encoding genes were deleted were also examined for virulence (**Figure 4C**). A decrease in virulence was observed in the Δ9-nonCTD-P chain mutant, but not in the Δ8-nonCTD-P mutant. The mutants described above were also examined for virulence against rainbow trout alevin (sac-fry). The mutant lacking 10-CTD-peptidase-encoding genes (Δ10-CTD-P) showed a decrease in virulence in rainbow trout alevin (**Figure 5**). Other CTD and nonCTD chain mutants tested caused similar mortality to wild type in alevin (data not shown).

### Defects in virulence are caused by the cumulative effect of deletion of multiple peptidase-encoding genes, including C6N29_13645

**Figure 6:**
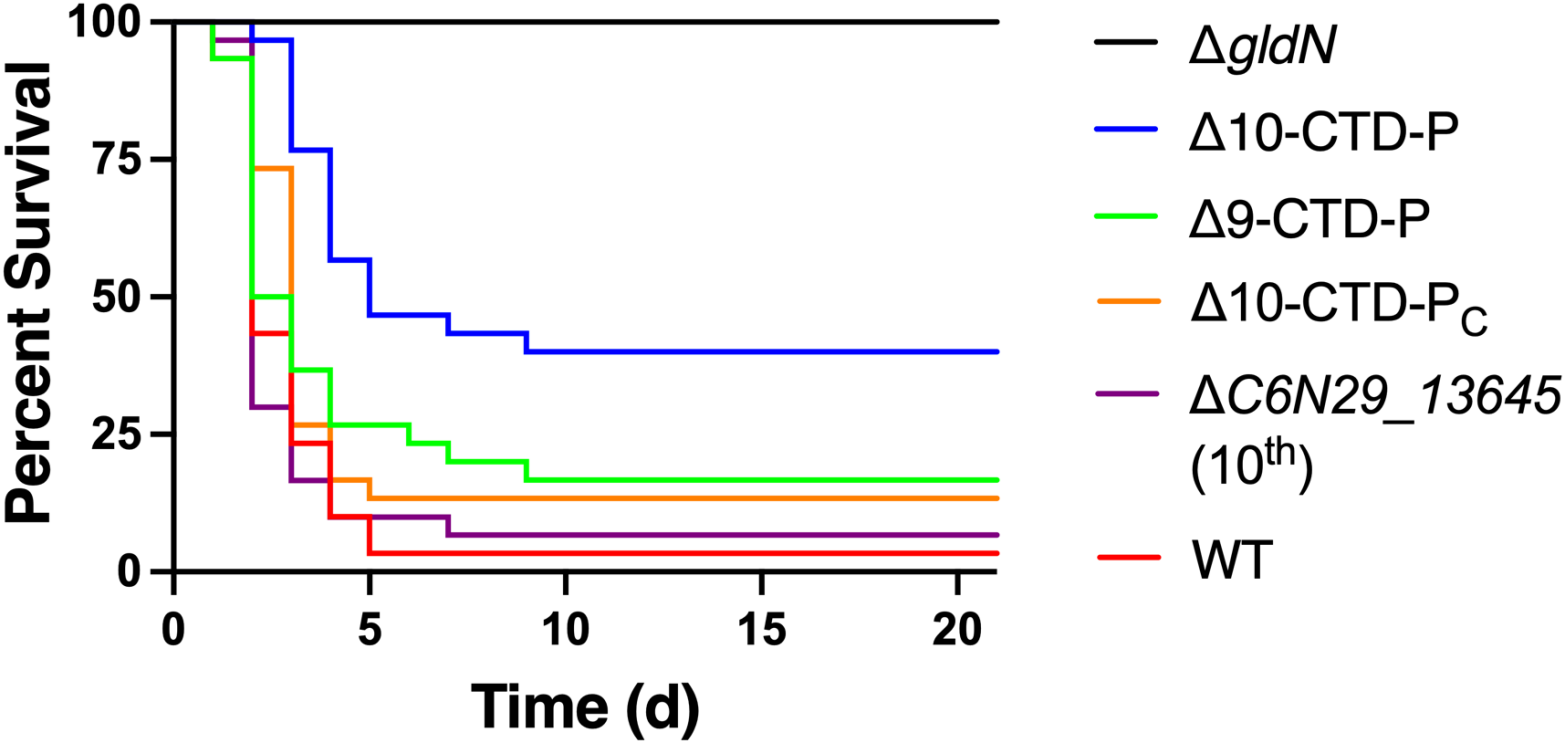
Effect of deletion of peptidase-encoding genes on virulence of *F. columnare* in juvenile rainbow trout. Rainbow trout were exposed by immersion to wild-type and mutant strains and survival was monitored for 21 days. Strains examined were wild-type *F. columnare* (WT); Δ*gldN*; Δ10-CTD-P; Δ9-CTD-P; ΔC6N29_13645; Δ10-CTD-P complemented by chromosomal insertion of C6N29_13645 (Δ10-CTD-P_C_). The final challenge concentrations were 7.6 × 10^6^ CFU/mL (WT), 9.3 × 10^6^ CFU/mL (Δ*gldN*), 7.1 × 10^6^ CFU/mL (Δ10-CTD-P), 7.3 × 10^6^ CFU/mL (Δ9-CTD-P), 7.6 × 10^6^ CFU/mL (ΔC6N29_13645), and 5.4 × 10^6^ CFU/mL (Δ10-CTD-P_C_). Significant differences in percent survival for fish challenged were observed between the WT and Δ10-CTD-P (*P*<0.0001).

A single deletion mutant lacking the 10^th^ CTD-peptidase gene (C6N29_13645) was examined for virulence in juvenile rainbow trout (**Figure 6**). ΔC6N29_13645 displayed similar virulence to wild type, indicating that deletion of the single gene, C6N29_13645, was not responsible for the defect in virulence observed in Δ10 CTD-P. Introduction of C6N29_13645 by genomic integration into the Δ10-CTD-P background restored virulence to wild-type levels. Thus, C6N29_13645 appears to be important for *F. columnare* virulence, but deletion of other peptidase-encoding genes and C6N29_13645 was necessary for defects in virulence.

### Deletion of *tspA*, encoding a predicted tail-specific protease, caused decreased virulence in rainbow trout and in adult zebrafish

**Figure 7:**
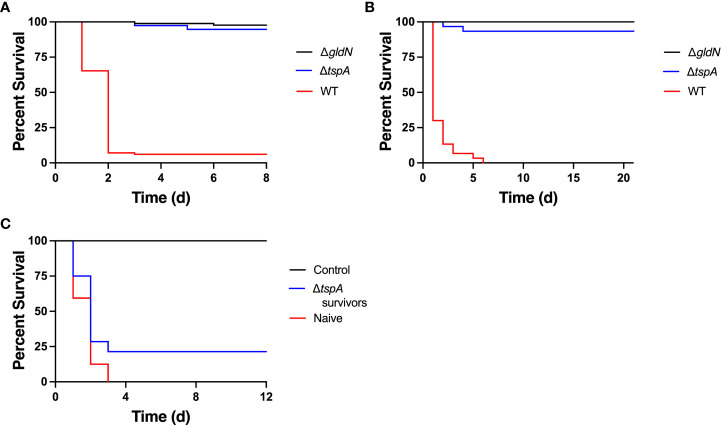
Effect of deletion of *tspA*, which encodes a tail-specific protease, on virulence. Strains examined were wild-type *F. columnare* (WT), Δ*tspA*, and Δ*gldN.* ***(*A*)*** Rainbow trout alevin were exposed by immersion to *F. columnare* and survival was monitored for 8 d. The final challenge concentrations were 6.9 × 10^6^ CFU/mL (WT), 1.1 × 10^7^ CFU/mL (Δ*gldN*), and 9.0× 10^5^ CFU/mL (Δ*tspA*). Significant differences in percent survival for fish challenged were observed between the WT and Δ*tspA* (*P*<0.0001). **(B)** Rainbow trout juveniles were exposed by immersion to *F. columnare* strains and survival was monitored for 21 d. The final challenge concentrations were 3.4 × 10^7^ CFU/mL (WT), 6.6 × 10^5^ CFU/mL (Δ*gldN*), and 9.6 × 10^6^ CFU/mL (Δ*tspA*). Significant differences in percent survival for fish challenged were observed between the WT and Δ*tspA* (*P*<0.0001). **(C)** Juvenile rainbow trout that survived exposure to Δ*tspA* (Δ*tspA* survivors) were examined for resistance to wild-type cells. Naïve fish and Δ*tspA* survivors were maintained for 28 d and then exposed to wild-type cells at 3.8 × 10^7^ CFU/mL. Survival was monitored for 12 d. Fish were exposed to TYES without bacteria as a control. Significant differences in percent survival for fish challenged were observed between naïve fish and Δ*tspA* survivors (*P*<0.05).

Deletion of C6N29_08680 (Δ*tspA*) caused decreased virulence in rainbow trout alevin, and rainbow trout juveniles, and adult zebrafish (**Figure 7** and **Figure S3**). Juvenile rainbow trout that survived exposure to Δ*tspA* were maintained for 28 days and then challenged with wild-type *F. columnare*. Previous exposure to Δ*tspA* did not protect most fish from later exposure to wild type *F. columnare* (**Figure 7C**).

### Growth of peptidase deletion mutants

**Figure 8:**
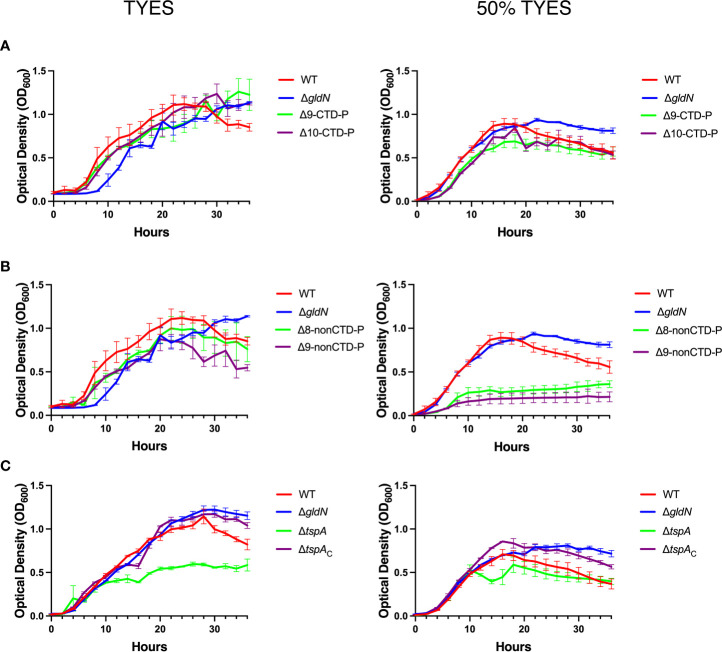
Growth curves for wild-type (WT) and mutant *F. columnare* strains. Cells were grown in TYES medium or 50% TYES medium at 28°C in a 48-well plate for 36 hours. Growth curves were performed in triplicate and error bars indicate standard error of the mean. **(A)** WT; Δ*gldN*; Δ9-CTD-P; Δ10-CTD-P. **(B)** WT; Δ*gldN*; Δ8-nonCTD-P; Δ9-nonCTD-P. Significant differences in bacterial cell density were observed in 50% TYES for Δ8-nonCTD-P and Δ9-nonCTD-P compared to the WT (*P*<0.0001). **(C)** WT; Δ*gldN*; Δ*tspA*, and Δ*tspA* complemented with pHM22 (Δ*tspA_c_*). Significant differences in bacterial cell density were observed in TYES for Δ*tspA* compared to the WT (*P* < 0.05).

Strains with severe defects in proteolytic activity or virulence were tested for differences in growth rate and survival compared to wild type (**Figure 8**). Mutants lacking peptidase genes with T9SS CTDs (**Figure 8A**) did not exhibit obvious growth defects compared to wild type. The T9SS-deficient mutant Δ*gldN* also grew as well as the wild type, as was also previously shown (Thunes et al., 2022). We also grew the strains in 50% TYES, which has half of the nutrients of the full-strength medium. In this medium the Δ*gldN* mutant unexpectedly outperformed the wild type at later time points, perhaps indicating greater stability of the mutant after cells reached stationary phase of growth.

The Δ8-nonCTD-P mutant grew similar to wild type in TYES (**Figure 8B**). In contrast, the Δ9-nonCTD-P mutant exhibited a growth defect, reaching a lower maximum optical density. In 50% TYES, both mutants exhibited growth defects compared to the wild type.

Deletion of the tail-specific protease gene, *tspA*, caused a growth defect in TYES (**Figure 8C**), resulting in decreased final optical density compared to the wild type. This defect was less noticeable in 50%TYES medium. The growth defect exhibited by this mutant may have contributed to the decreased virulence that was observed for cells grown in TYES and used to challenge rainbow trout (**Figure 7**).

## Discussion

*F. columnare* causes high mortality and large economic losses in freshwater aquaculture. Although the T9SS has been implicated in columnaris disease (Li et al., 2017; Thunes et al., 2022), little is known regarding which of the many secreted proteins impact virulence. Secreted proteases have previously been suggested as virulence factors of *F. columnare* (Bertolini and Rohovec, 1992; Newton et al., 1997; Declercq et al., 2013). Disruption of the T9SS by deletion of *gldN* or *porV* greatly reduces extracellular proteolytic activity and eliminates virulence (Li et al., 2017; Thunes et al., 2022). Here we selected 20 of 38 identified genes encoding secreted peptidases for deletion to determine if any are critical for virulence. We focused only on those peptidases that were present in cell-free spent TYES medium collected from wild-type cultures at mid-log phase, and that were secreted at much lower level if at all by the avirulent and T9SS-defective Δ*gldN* mutant (Thunes et al., 2022). The wild-type cells used in our fish challenge experiments were grown to the same growth phase in TYES, so it is likely that they also produced and secreted these peptidases.

The expression of so many peptidases makes functional redundancy likely. Because of this we constructed CTD-peptidase and nonCTD-peptidase chain mutants lacking multiple peptidases. Mutants from both deletion chains exhibited defects in extracellular proteolysis. Defects in extracellular proteolytic activity similar to those of the Δ*gldN* mutant were observed for mutants in both chains that lacked nine or ten protease-encoding genes. Investigation of single gene deletions and complemented strains suggested that the source of the proteolysis defects in the CTD chain mutant was a cumulative effect of deletion of multiple genes. However, deletion of the single nonCTD peptidase gene C6N29_03570 also resulted in a significant reduction in proteolysis. Surprisingly, deletion of one peptidase-encoding gene (C6N29_14605) from a strain lacking seven other peptidase-encoding genes resulted in increased levels of proteolysis (**Figure 2**, Δ7-nonCTD-P). We do not know the reason for this, but one possibility is that deletion of the peptidase-encoding gene caused a regulatory effect that increased expression of other peptidases.

In challenges of larval and adult zebrafish, chain mutants lacking up to 10 CTD-peptidase genes, and up to nine nonCTD-peptidase genes were as virulent as the wild type. Similarly in rainbow trout fry, chain mutants lacking up to nine CTD-peptidase genes, and up to eight nonCTD-peptidase genes were fully virulent. However, a mutant lacking 10 CTD-peptidase genes and a mutant lacking nine nonCTD-peptidase genes exhibited partial defects in virulence for rainbow trout fry, and the Δ10-CTD-P mutant also exhibited decreased virulence in rainbow trout alevin. Virulence was restored to the Δ10-CTD-P mutant by introduction of C6N29_13645, the 10^th^ CTD-peptidase gene that had been deleted in the Δ10-CTD-P chain mutant. However, a mutant lacking just C6N29_13645 was fully virulent. Together, these results suggest that C6N29_13645 is important for virulence, but that the decreased virulence observed for the Δ10-CTD-P mutant resulted from the cumulative effect of deletion of C6N29_13645 and other peptidase-encoding genes. This suggests partial redundancy of function of some peptidases involved in virulence.

Previously, a mutant lacking three genes, the CTD-peptidase gene C6N29_05800 and two nonCTD-peptidase genes, C6N29_11545 and C6N29_11550, displayed a decrease in virulence in rainbow trout fry compared to wild type (Thunes et al., 2022). In contrast, in the current study mutants lacking these genes appeared to be fully virulent. This discrepancy may be the result of differences in the challenge doses used in the different studies. Regardless, in both studies the absence of these three genes did not result in avirulence.

Unlike other mutants lacking single peptidase-encoding genes, deletion of *tspA*, which encodes a predicted tail-specific protease, caused decreased mortality in adult zebrafish, juvenile rainbow trout, and rainbow trout alevin. In contrast, deletion of *tspA* did not cause a noticeable decrease in extracellular proteolytic activity. This is not surprising because as a bacterial tail-specific protease, TspA is expected to process specific regions of select bacterial proteins rather than to function as a general protease. We do not know the function of TspA, but it likely impacts the functions of the specific proteins that it processes. The Δ*tspA* mutant exhibited a growth defect in the rich growth medium, TYES. We do not know the reason for this growth defect, but it may explain the decreased virulence of this mutant. In other bacteria, mutations in genes encoding tail-specific proteases have resulted in defects in growth, virulence, and sensitivity to various stresses (Saoud et al., 2021; Sommerfield and Darwin, 2022).

Juvenile rainbow trout that survived exposure to Δ10-CTD-P were not protected against later exposure to the wild type. These mutants may not have mounted enough of an infection to result in a strong immune response. Alternatively, the peptidases that were absent in the mutant may have been protective antigens, and in their absence, a protective immune response may not occur. Further study of mutants lacking secreted proteins may identify some that are attenuated for virulence but that generate protective immune responses.

The Δ10-CTD-P gene deletion mutant exhibited decreased virulence in rainbow trout fry, but not in adult or larval zebrafish. There are many differences between zebrafish and rainbow trout, and between the challenge protocols used, that may account for these results. For example, zebrafish are warm-water fish, whereas rainbow trout are cold-water fish. Unlike some other temperature-dependent aquatic pathogens (Tang et al., 2020), *F. columnare* can infect different hosts at different temperatures. The temperatures used in the challenges (26°C to 28°C for zebrafish and 16°C for rainbow trout) may have altered *F. columnare* gene expression, enzyme activity, or enzyme stability, affecting virulence. Adult or larval zebrafish and juvenile rainbow trout may also have differences in their innate resistance to *F. columnare* strains.

Peptidases have been suggested as virulence factors for *F. columnare* and related *Flavobacterium* fish pathogens (Declercq et al., 2013). Deletion of a pair of *F. psychrophilum* peptidase-encoding genes resulted in decreased proteolytic activity but resulted in no significant defect in virulence (Pérez-Pascual et al., 2011). This mimics many of the results presented here for *F. columnare*. Only the Δ*tspA*, Δ10-CTD-P, and Δ9-nonCTD-P mutants displayed reduced virulence in rainbow trout. Peptidases appear to be important for virulence, but understanding their exact roles requires further study. We deleted many genes encoding predicted peptidases, but our study was not exhaustive. Other *F. columnare* peptidase-encoding genes that were not examined here could also contribute to virulence.

The results presented here indicate that secreted CTD-peptidases and nonCTD-peptidases contribute to virulence. Some peptidases have significant effects individually on virulence, some appear to be redundant, and others may have little if any effect on virulence. Further studies are needed to fully elucidate which of the many peptidases are most critical, and under what conditions they are expressed. Such proteins could be targets for therapeutics or vaccine development.

## Methods

### Bacterial strains and growth conditions

*F. columnare* wild-type strain MS-FC-4 (Evenhuis and LaFrentz, 2016; Bartelme et al., 2018) and mutants derived from this strain were grown at 28°C for liquid cultures, and at 30°C for agar cultures, in tryptone yeast extract salts (TYES) medium as previously described (Cain and LaFrentz, 2007; Thunes et al., 2022). *F. columnare* cultures used for rainbow trout challenges were grown in TYES-2xMg, which is identical to TYES except that it contains twice as much MgSO_4_. *E. coli* strains were grown at 37°C in lysogeny broth (LB) (Bertani, 1951). The strains and plasmids used in this study are listed in **Table S2**, and primers are listed in **Table S3**. Antibiotics were used in the following concentrations unless otherwise specified: 100 µg/mL ampicillin, 1 µg/mL tetracycline, and 1 µg/mL tobramycin.

### Conjugative transfer of plasmids into *F. columnare*

Plasmids were transferred from *E. coli* S17-1 λ pir into *F. columnare* strain MS-FC-4 by conjugation as previously described (Thunes et al., 2022).

### Construction of deletion mutants

In-frame gene deletion mutants were constructed as previously described (Li et al., 2015; Li et al., 2017; Thunes et al., 2022). These deletions leave upstream and downstream regions unaltered to limit the possibility of polar effects on downstream genes. To delete C6N29_05315, a 2.2 kbp region upstream of C6N29_05315 was amplified by PCR using Phusion DNA polymerase (New England Biolabs, Ipswich, MA) and primers 2270 (adding a KpnI site) and 2271 (adding a XbaI site). The product was digested with KpnI and XbaI and ligated into pMS75 that had been digested with the same enzymes, to produce pNT23. A 2.5 kbp region downstream of C6N29_05315 was amplified using primers 2272 (adding a XbaI site) and 2273 (adding a PstI site). The product was digested with XbaI and PstI and ligated into pNT23 that had been digested with the same enzymes, to generate pNT32. Plasmid pNT32 was transferred to *F. columnare* MS-FC-4 by conjugation, and colonies with the plasmid recombined into the chromosome were obtained by selecting for tetracycline resistance. Colonies were streaked for isolation on TYES containing 5 µg/mL tetracycline and isolated colonies were grown in liquid without tetracycline to allow for plasmid loss. The cells were plated on TYES media containing 5% sucrose and the mutant was obtained by selecting for sucrose resistance. PCR was performed to confirm the deletion. The other gene deletion mutants were constructed in the same way, using the primers listed in **Table S3** to obtain the appropriate upstream and downstream regions to construct the plasmids listed in **Table S2**.

### Plasmid complementation of mutants

A 1.3 kbp fragment spanning C6N29_12115 was amplified using Phusion DNA polymerase and primers 2535 (adding a KpnI site) and 2536 (adding a SphI site). The product was digested with KpnI and SphI and ligated into the shuttle vector pCP23, which was digested with the same enzymes, to produce pHM23. pHM23 was transferred into the *F. columnare* C6N29_12115 deletion mutant by conjugation. Plasmid complementation of other mutants was conducted in the same way, using the plasmids listed in **Table S2**, and the primers listed in **Table S3**.

### Complementation of mutants by chromosomal insertion

A 7.4 kbp fragment spanning C6N29_13645 and 2 kbp regions upstream and downstream was amplified using Q5 DNA Polymerase (New England Biolabs, Ipswich, MA) and primers 2298 (adding a KpnI site) and 2301 (adding a PstI site). The product was digested with KpnI and PstI and ligated into the suicide vector pMS75, which was digested with the same enzymes, to produce pNT76. pNT76 was transferred into the *F. columnare* C6N29_13645 deletion mutant by conjugation. Colonies containing pNT76 recombined into the chromosome were selected using tetracycline resistance (5 µg/mL tetracycline). These were then grown without antibiotic to allow plasmid loss by a second recombination event, and plated on media containing sucrose to eliminate cells that retained the integrated plasmid. Sucrose resistant colonies were screened using PCR to confirm the reinsertion of C6N29_13645 into its native site.

### Growth curves

Growth curves were performed as previously described with slight modifications (Thunes et al., 2022). *F. columnare* cultures were streaked from freezer stocks onto TYES agar and incubated for 24 h at 30°C. These cultures were used to inoculate 50 mL of TYES broth and were incubated 14 h at 28°C shaking at 200 rpm. Cultures were standardized to an OD_600_ of 0.5 and used to inoculate wells in a 48-well polystyrene plate. 50 µL of culture and 950 µL of TYES were added to each well. All cultures were analyzed in triplicate using TYES as a negative control and blank. Plates were incubated at 28°C with shaking at 200 rpm and readings were taken at two-hour intervals for 36 h using a CLARIOstar Microplate Reader (BMG Labtech, Ortenberg, Germany).

### Proteolytic activity

Proteolytic activity was quantified using azocasein as a substrate as previously described (Thunes et al., 2022), except that the *F. columnare* cultures were grown for two additional hours before analysis. In particular, strains were incubated in 50 mL TYES broth overnight at 28°C with shaking at 200 rpm. These overnight cultures were standardized to an OD_600_ of 0.5 and then 120 µL was used to inoculate 6 mL TYES broth. Triplicate cultures were incubated at 28°C with shaking at 200 rpm for 22 h (Thunes et al., 2022). Cells were removed by centrifugation and the supernatant was filtered to result in cell-free spent culture fluid. Protein concentration was determined for bacterial cell pellets, and protease activity was determined on the cell-free spent culture fluid as described (Thunes et al., 2022).

### Challenges of adult zebrafish

Zebrafish (*Danio rerio*) challenges were performed as previously described (Thunes et al., 2022), with minor modifications as indicated below. *F. columnare* wild-type, mutant, and complemented mutant strains were grown in TYES overnight at 28°C. 5 ml of overnight culture was diluted into 25 mL of fresh TYES and grown until OD_600_ reached 0.5. To determine the number of live cells per mL, cultures were serially diluted and plated on TYES agar. To test the virulence of each strain, naïve adult Eckwill zebrafish were immersed in a solution of 0.5 ml *F. columnare* cells and 99.5 mL dechlorinated Milwaukee municipal tap water for 30 min at 26°C. Control fish were exposed to 0.5 mL growth medium without *F. columnare* in 99.5 mL of water. After exposure fish were moved to tanks with 2 L fresh water at 28°C and observed for up to ten days for signs of infection. Each treatment was performed in triplicate tanks, with each tank containing five zebrafish in 2 L water. Mortalities were recorded daily. A minimum of 20% of the fish that died were examined for the presence of bacteria phenotypic of *F. columnare* (yellow, rhizoid, tobramycin resistant colonies) by swabbing gills, fins, and skin, streaking on TYES agar containing tobramycin, and incubating for 2 d at 30°C. *F. columnare* was detected in all mortalities tested. No signs of disease were observed prior to challenge and no indications of *F. columnare* or columnaris disease were observed in the uninfected control tanks or in the maintenance tanks at any time.

In some cases, survivors of challenges with *F. columnare* mutants were examined for resistance to later infection by wild-type *F. columnare*. For these experiments, fish were maintained in 2 L of water for 28 d after the initial exposure to the mutant strain. Some of the fish were then challenged with the wild type, as described above, and others were exposed to the same volume of TYES medium without bacteria. Naïve fish were also challenged with the wild type at the same time.

### Challenge of germ-free zebrafish larvae

Germ-free zebrafish larvae were challenged with wild-type, mutant, and complemented strains of *F. columnare* strain MS-FC-4 at 28°C as previously described (Stressmann et al., 2021). Briefly, 10 to 12 germ-free larvae (6 days postfertilization) were exposed to 10^4^ colony-forming-units (CFU)/mL of washed *F. columnare* cells for 3 hours in 25-cm^3^ culture flasks with vented caps containing 20 mL of sterile mineral water. They were then transferred individually to 24-well plates containing 2 mL sterile water per well. Larvae were fed every 48 h with 50 µL of germ-free *Tetrahymena thermophila* per well. Mortalities were counted daily and measured in days-post-infection (dpi) with 0 dpi corresponding to the infection day. All zebrafish larval experiments were stopped at 9 dpi, and zebrafish were euthanized with tricaine (MS-222) (Sigma-Aldrich; catalog no. E10521). Each experiment was repeated at least twice.

### Rainbow trout challenges

Rainbow trout (*Oncorhynchus mykiss*) challenges for juveniles and alevin were performed using rainbow trout reared from certified disease-free eggs from Troutlodge Inc., Sumner, WA. Trout were maintained as described previously (Thunes et al., 2022) at the USDA-ARS National Center for Cool and Cold Water Aquaculture research facility in Kearneysville, WV in flow through water at a rate of 1 L/min, at 12.5°C, until the challenge weight of ~1.3 g was met. The fish in this facility are checked yearly for multiple diseases including columnaris disease, and except for fish in the challenge room, they are certified disease-free. No signs of disease were observed prior to challenge and no indications of *F. columnare* or columnaris disease were observed in the uninfected control tanks or in the maintenance tanks at any time. Fish were moved to challenge aquaria 1 week prior to immersion challenge to acclimate to the elevated water temperature of 16°C.

Wild-type, mutant and complemented *F. columnare* strains were each used for immersion challenges. Frozen bacterial stocks were stored at -80°C in 75% TYES-2xMg broth and 25% glycerol. Bacterial cultures, for challenges, were grown as previously described (Evenhuis and LaFrentz, 2016; Conrad et al., 2022a). Briefly, cultures were incubated at 30°C with shaking at 150 rpm until OD_540_ of 0.5 to 0.6 was reached, at which point the cells were used for the challenge.

Challenges of fry were performed using triplicate 3 L tanks with restricted water flows (~200 mL/min) at 16°C. Each tank contained 40 fish of approximately 1.35 g each. Water flows were stopped for the immersion challenge and tanks were inoculated with bacterial cultures and incubated for 0.5 h after which water flows were resumed. Control tanks were inoculated with sterile TYES-2xMg broth. Serial dilutions of water samples from each tank after inoculation were plated on TYES-2xMg agar to determine CFU/mL. Mortalities were removed and counted daily. The data for triplicate tanks of each strain were pooled and survivor fractions for each strain were calculated. Challenges continued for 21 days or until 3 days without recorded mortalities post-exposure. Approximately 16% of mortalities were randomly tested by homogenizing gill tissue and streaking on TYES-2xMg agar plates to determine if *F. columnare* was present. Confirmation of *F. columnare* was determined by morphological observation of yellow, rhizoid, adherent colonies and by amplifying 16S rRNA genes and confirming the genomovar by enzymatic digestion (HaeIII) and gel electrophoresis as previously described (Triyanto and Wakabayashi, 1999; Olivares-Fuster et al., 2007; LaFrentz et al., 2014). *F. columnare* was detected in all mortalities tested and all were genomovar I and genetic group 1, as expected for strain MS-FC-4.

Challenges of rainbow trout alevin were performed as previously described (Evenhuis et al., 2021). In brief, 100 alevin were challenged 3 days post-hatch in 3-liter tanks. Total CFUs are given in the figures. Mortalities were removed daily and whole alevin were homogenized and streaked on TYES agar plates to determine the presence of *F. columnare*.

### Bioinformatic analyses

Genome sequences were analyzed for T9SS genes encoding proteins that belong to appropriate TIGRFAM multiple-sequence alignment families (Haft et al., 2013). The genomes were also examined for genes encoding predicted secreted proteins that have type A CTDs (TIGR04183) and type B CTDs (TIGR04131 and pfam13585) (Veith et al., 2017). In each case, the trusted cutoffs assigned by The J. Craig Venter Institute (JCVI) that allow identification of the vast majority of family members with vanishingly few false positives (Haft et al., 2013) were used. Some bioinformatic data were taken from (Thunes et al., 2022). Predicted *F. columnare* T9SS peptidases listed in **Table 2** and **Table S1** were identified by BLASTP analyses, and by using resources on the MEROPS website (https://www.ebi.ac.uk/merops/) (Rawlings et al., 2018) and the National Center for Biotechnology Information (NCBI) conserved domain searches (Marchler-Bauer et al., 2015; Marchler-Bauer et al., 2017).

### Statistical analyses

A value of *p* < 0.05 was considered significant. For characterization assays a one-way ANOVA with Tukey’s post-test was used to analyze differences between treatment groups, unless otherwise noted. Error bars represent SEM (standard error of the mean), except for **Figure 3**, where they represent standard deviation. Kaplan-Meier survival analyses (Kaplan and Meier, 1958) were performed on fish challenge data. GraphPad Prism version 9.4.1 (GraphPad Software, LLC) was used to compute statistical tests.

## Data availability statement

The original contributions presented in the study are included in the article/**Supplementary Material**. Further inquiries can be directed to the corresponding author.

## Ethics statement

The animal study was reviewed and approved by the University of Wisconsin-Milwaukee Institutional Animal Care and Use Committee (adult zebrafish studies), the Institut Pasteur institutional Animal Health and Care Committees under permit # dap200043 (zebrafish larvae studies), and the US Department of Agriculture NCCCWA Institutional Animal Care and Use Committee (rainbow trout challenges, as described in Protocol #176).

## Author contributions

NT and MM conceived and designed the study and performed the bioinformatic analyses. MM, JE, and J-MG obtained funding to support the research. NT and HM constructed all bacterial mutants and performed challenge experiments on adult zebrafish. RC constructed two plasmids used in mutant construction. D-PP, RS and J-MG performed and analyzed all experiments on zebrafish larvae. JE, RL, and CB performed all experiments involving rainbow trout. NT performed and analyzed growth experiments and protease assays, and analyzed adult zebrafish and rainbow trout challenge data. NT wrote the initial draft of the manuscript. NT, MM, HM, D-PP, RS, JE, J-MG and RL contributed to manuscript revision. All authors approved the submitted version.
