# Supplementary material for: Secreted peptidases contribute to virulence of fish pathogen Flavobacterium columnare
Source: Front Cell Infect Microbiol. 2023 Feb 3;13:1093393. doi: 10.3389/fcimb.2023.1093393 (PMC9936825; doi:10.3389/fcimb.2023.1093393)
Supplement: Supplementary file 1 [file DataSheet_1.pdf]

Supplemental Figures and Tables for:

Nicole C. Thunes, Haitham H. Mohammed, Jason P. Evenhuis, Ryan S. Lipscomb, David Pérez-Pascual, Rebecca J. Stevick, Clayton Birkett, Rachel A. Conrad, Jean-Marc Ghigo, Mark J. McBride. 2023. Secreted peptidases contribute to virulence of fish pathogen *Flavobacterium columnare*. **Front. Cell. Infect. Microbiol**. **13**:1093393. doi: 10.3389/fcimb.2023.1093393

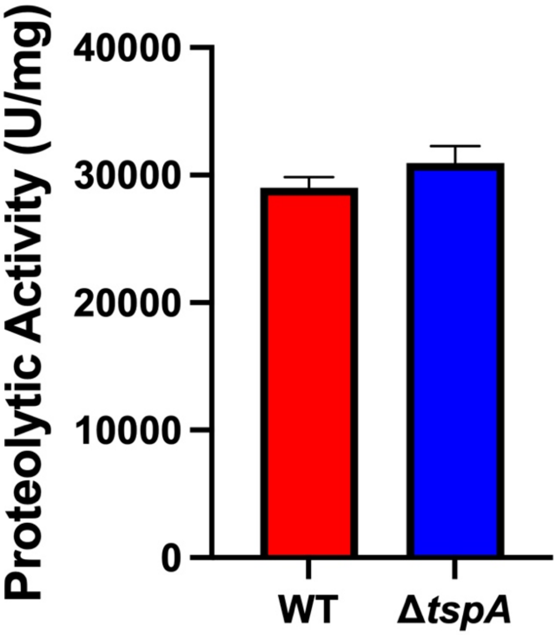

Figure S1. Secreted proteolytic activities of wild type and ΔC6N29_08680 (Δ*tspA*). Statistics correspond to one-way ANOVA with Tukey post-test comparing Δ*tspA* to wild type and revealed no significant difference.

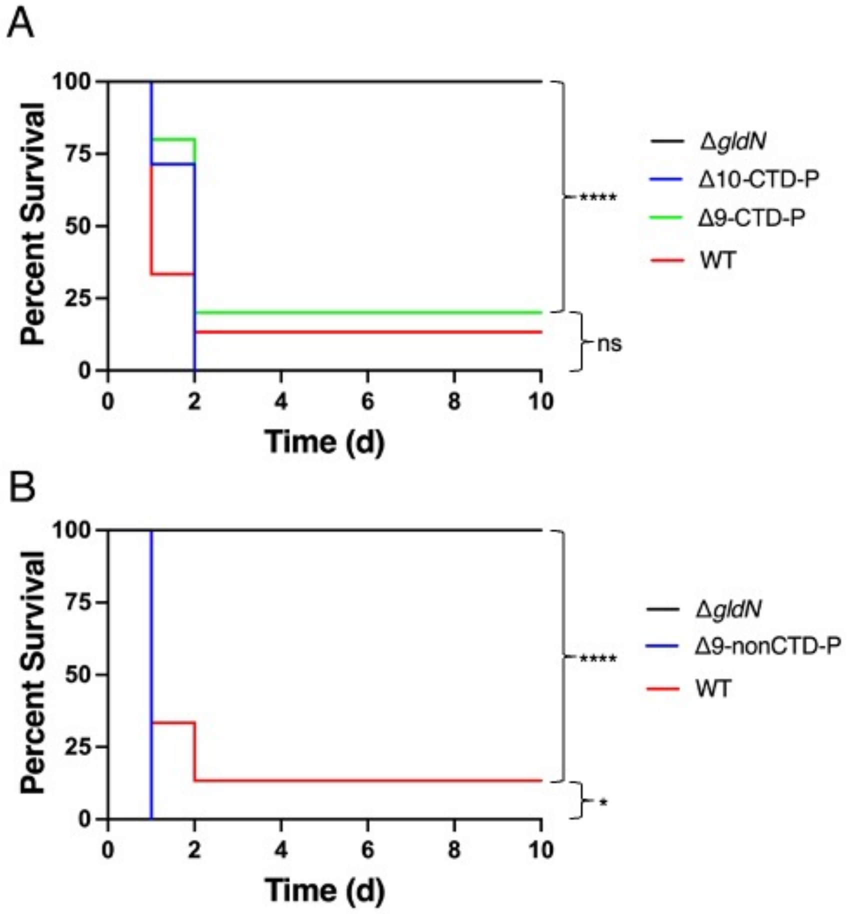

Figure S2. Effect of deletion of peptidase-encoding genes on virulence in adult zebrafish. Zebrafish were exposed by immersion to *F. columnare* strains for 30 min at 26°C, transferred to fresh water, and percent survival was monitored for 10 d. Strains examined were **(A)** wild-type (WT); Δ*gldN* mutant; Δ10-CTD-P; Δ9-CTD-P; **(B)** WT; Δ*gldN* mutant; Δ9-nonCTD-P. The final challenge concentrations were 1.4×10^6 CFU/mL (WT), 2.8×10^6 CFU/mL (Δ*gldN*), 1.2×10^6 CFU/mL (Δ10-CTD-P); 1.1×10^6 CFU/mL (Δ9-CTD-P), 1.1×10^6 CFU/mL (Δ9-nonCTD-P). Fifteen fish were challenged with each strain as indicated in Methods. Kaplan-Meier survival analyses (Kaplan and Meier, 1958) were performed using GraphaPad Prism. ns, not significant; *, $p<0.05$; ****, $p<0.0001$.

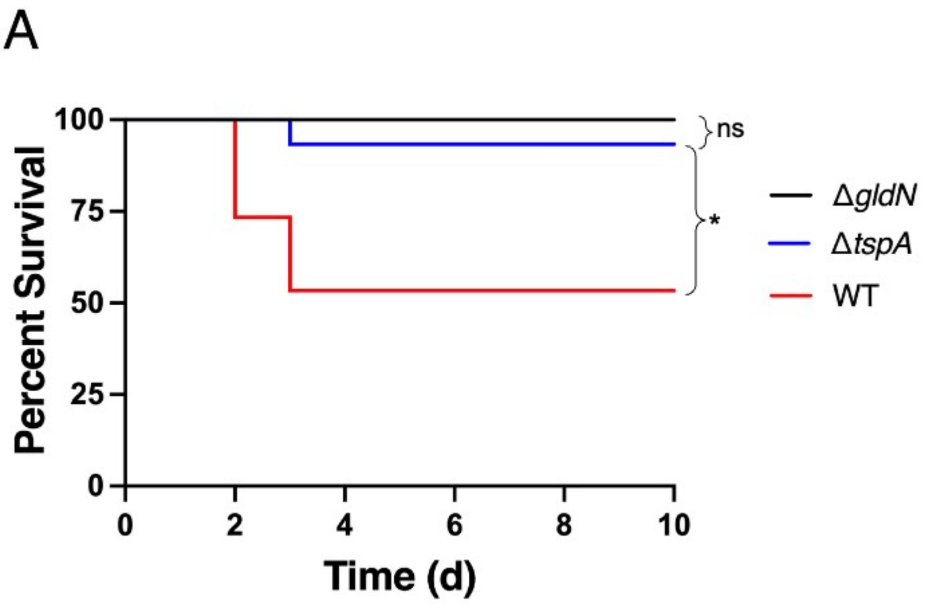

Figure S3. Effect of deletion of *tspA*, which encodes a tail-specific protease, on virulence in adult zebrafish. Adult zebrafish were exposed by immersion to *F. columnare* strains for 30 min at 26ºC, transferred to fresh water, and survival was monitored for 10 d. The final challenge concentrations were 7.9×10^5 CFU/mL (WT), 2.0×10^6 CFU/mL (Δ*gldN*), and 2.9×10^6 CFU/mL (Δ*tspA*). n = 15. Kaplan-Meier survival analyses (Kaplan and Meier, 1958) were performed using GraphPad Prism. ns, not significant; *, $p<0.05$.

Table S1. Secreted *F. columnare* MS-FC-4 peptidases[a]

**Secreted *F. columnare* MS-FC-4 Peptidases**				**Total Spectral Counts**		
**Locus Tag**	**Protein ID**	**Predicted Function**	**Identified T9SS CTD**	**Wild Type**	**gldN Mutant**	**gldN Compl**
C6N29_09900	PTD14721.1	possible peptidase	Yes	120	0	20
C6N29_10620	PTD14860.1	subfamily M43B peptidase	No	104	10	89
C6N29_05315	PTD13902.1	subfamily S8A peptidase	Yes	94	0	92
C6N29_00585	PTD16151.1	S9 family peptidase	No	61	0	49
C6N29_11545	PTD15009.1	metalloprotease	No	61	1	43
C6N29_01145	PTD16253.1	peptidase M16	No	52	8	63
C6N29_12115	PTD15115.1	zinc metalloprotease	No	46	0	32
C6N29_01770	PTD16368.1	peptidase M61	No	42	6	45
C6N29_14605	PTD15568.1	S9 family peptidase	No	41	0	35
C6N29_07780	PTD14344.1	possible subtilisin-like protease	Yes	37	0	29
C6N29_11550	PTD15010.1	metalloprotease	No	37	0	17
C6N29_10335	PTD14805.1	probable peptidase	Yes	35	0	14
C6N29_03570	PTD13591.1	S46 family peptidase	No	34	0	24
C6N29_08730	PTD14510.1	S9 family peptidase	No	32	0	26
C6N29_13645	PTD15387.1	M4 family peptidase	Yes	28	0	3
C6N29_08590	PTD14484.1	subfamily M48C peptidase	No	26	17	27
C6N29_12020	PTD15097.1	zinc metalloprotease	No	24	0	22
C6N29_10010	PTD15738.1	peptidase C1A papain	No	22	0	6
C6N29_04605	PTD13770.1	probable peptidase	Yes	21	0	13
C6N29_03390	PTD13559.1	probable peptidase	Yes	20	0	9
C6N29_08680	PTD14500.1	tail-specific peptidase	No	19	0	13
C6N29_05800	PTD13986.1	probable peptidase	Yes	19	0	8
C6N29_10910	PTD14907.1	probable peptidase	Yes	19	0	20
C6N29_06855	PTD14169.1	subfamily M20F dipeptidase	No	18	0	21
C6N29_08145	PTD14409.1	probable metallopeptidase	Yes	17	0	5
C6N29_09865	PTD14714.1	probable peptidase	Yes	17	1	15
C6N29_04610	PTD13771.1	S9 family peptidase	No	16	1	22
C6N29_06420	PTD14090.1	S41 family peptidase	No	12	0	5
C6N29_04960	PTD13840.1	peptidase M1	No	10	0	13
C6N29_11805	PTD15055.1	peptidase M28	No	7	0	2
C6N29_09870	PTD14715.1	peptidase M28	No	6	0	5
C6N29_05910	PTD14008.1	S9 family peptidase	No	5	0	5
C6N29_02905	PTD16572.1	PorU; T9SS C-terminal processing peptidase	Yes	4	0	4
C6N29_07280	PTD15722.1	peptidoglycan endopeptidase	No	3	0	0
C6N29_03470	PTD13575.1	aminopeptidase	No	2	0	1
C6N29_11100	PTD14931.1	serine protease	No	2	0	0
C6N29_00495	PTD16135.1	family peptidase	No	2	0	0
C6N29_00665	PTD16165.1	peptidase M1	No	2	0	1
C6N29_02875	PTD16628.1	peptidase M28	No	2	0	3

[a] Peptidases sorted by number of total LC-MS/MS spectral counts from cell-free spent culture fluid from wild-type cells as reported in Dataset S1 of Thunes et al 2022 (Thunes et al., 2022). Spectral counts from that study are also shown for the T9SS-deficient Δ*gldN* mutant and for the complemented Δ*gldN* mutant. Blue indicates peptidase-encoding genes with T9SS CTDs that were deleted in this study. Yellow indicates peptidase-encoding genes without clear T9SS CTDs that were deleted in this study. Lack of highlighting indicates genes not deleted in this study.

Table S2. Strains and plasmids used in this study

Strain or plasmid	Description[a]	Source or reference
E. coli **strains**		
DH5αMCR	Strain used for general cloning	Life Technologies (Grand Island, NY)
S17-1 λ pir	Strain used for conjugation	(de Lorenzo and Timmis, 1994)
F. columnare **strains (all derived from the wild type strain MS-FC-4)**		
MS-FC-4	Wild type	(Evenhuis and LaFrentz, 2016; Bartelme et al., 2018)
FCB14	Δ*gldN*; deletion of T9SS and motility gene C6N29_09600	(Thunes et al., 2022)
FCB20	Δ1-CTD-P; deletion of C6N29_05800 encoding predicted peptidase with T9SS CTD.	(Thunes et al., 2022)
FCB45	Δ(C6N29_11545 and C6N29_11550); deletion of two genes encoding predicted peptidases without clear CTDs.	(Thunes et al., 2022)
FCB54	Δ2-nonCTD-P; Δ(C6N29_11545 and C6N29_11550) ΔC6N29_05800; deletion of two genes encoding predicted peptidases without clear CTDs and one predicted peptidase with a T9SS CTD.	(Thunes et al., 2022)
FCB98	Δ2-CTD-P; deletion of C6N29_05315 encoding predicted peptidase in FCB20; deletion of two genes encoding predicted peptidases with T9SS CTDs.	This study
FCB100	Δ3-nonCTD-P; deletion of C6N29_00585 encoding predicted peptidase in FCB54; deletion of three genes encoding predicted peptidases without clear CTDs and one predicted peptidase with a T9SS CTD. Constructed by HM	This study
FCB101	Δ4-nonCTD-P; deletion of C6N29_08590 encoding predicted peptidase in FCB100; deletion of four genes encoding predicted peptidases without clear CTDs and one predicted peptidase with a T9SS CTD. Constructed by HM.	This study
FCB102	Δ5-nonCTD-P; deletion of C6N29_12020 encoding predicted peptidase in FCB101; deletion of two five encoding predicted peptidases without clear CTDs and one predicted peptidase with a T9SS CTD. Constructed by HM.	This study
FCB103	Δ6-nonCTD-P; deletion of C6N29_14605 encoding predicted peptidase in FCB102; deletion of six genes encoding predicted peptidases without clear CTDs and one predicted peptidase with a T9SS CTD. Constructed by HM.	This study
FCB105	Δ3-CTD-P; deletion of C6N29_07780 encoding predicted peptidase in FCB98; deletion of three genes encoding predicted peptidases with T9SS CTDs	This study
FCB107	Δ7-nonCTD-P; deletion of C6N29_03570 encoding predicted peptidase in FCB103; deletion of seven genes encoding predicted peptidases without clear CTDs and one predicted peptidase with a T9SS CTD. Constructed by HM.	This study

FCB109	Δ4-CTD-P; deletion of C6N29_10335 encoding predicted peptidase in FCB105; deletion of four genes encoding predicted peptidases with T9SS CTDs	This study
FCB117	Δ8-nonCTD-P; deletion of C6N29_12115 encoding predicted peptidase in FCB107; deletion of eight genes encoding predicted peptidases without clear CTDs and one predicted peptidase with a T9SS CTD. Constructed by HM.	This study
FCB134	Δ5-CTD-P; deletion of C6N29_10910 encoding predicted peptidase in FCB109; deletion of five genes encoding predicted peptidases with T9SS CTDs. Constructed by HM.	This study
FCB135	Deletion of C6N29_08680 (Δ*tspA*) encoding predicted tail-specific protease.	This study
FCB137	Δ9-nonCTD-P; deletion of C6N29_06855 encoding predicted peptidase in FCB117; deletion of nine genes encoding predicted peptidases without clear CTDs and one predicted peptidase with a T9SS CTD. Constructed by HM.	This study
FCB139	Δ6-CTD-P; deletion of C6N29_04605 encoding predicted peptidase in FCB134; deletion of six genes encoding predicted peptidases with T9SS CTDs	This study
FCB142	Δ7-CTD-P; deletion of C6N29_03390 encoding predicted peptidase in FCB139; deletion of seven genes encoding predicted peptidases with T9SS CTDs	This study
FCB145	Δ8-CTD-P; deletion of C6N29_09865 encoding predicted peptidase in FCB142; deletion of eight genes encoding predicted peptidases with T9SS CTDs	This study
FCB147	Δ8th NonCTD-P; ΔC6N29_12115 encoding predicted peptidase without clear CTD. Constructed by HM.	This study
FCB155	Δ9-CTD-P; deletion of C6N29_08145 encoding predicted peptidase in FCB145; deletion of nine genes encoding predicted peptidases with T9SS CTDs	This study
FCB159	Δ9th NonCTD-P; ΔC6N29_06855 encoding predicted peptidase without clear CTD. Constructed by HM.	This study
FCB167	Δ10-CTD-P; deletion of C6N29_13645 encoding predicted peptidase in FCB155; deletion of ten genes encoding predicted peptidases with T9SS CTDs	This study
FCB222	Δ9th CTD-P; ΔC6N29_08145 encoding predicted peptidase with T9SS CTD	This study
FCB230	Δ10th CTD-P; ΔC6N29_13645 encoding predicted peptidase with T9SS CTD	This study
FCB237	Δ10-CTD-P_C; wild-type C6N29_13645 restored to the native site of Δ10 CTD-P by chromosomal insertion with pNT76.	This study
FCB238	ΔC6N29_13645$_C$; wild-type C6N29_13645 restored to the native site of ΔC6N29_13645 by chromosomal insertion with pNT76.	This study
**Plasmids**		
pCP23	*E. coli-F. columnare* shuttle plasmid; Ap^r (Tc^r)	(Agarwal et al., 1997)
pMS75	Suicide vector carrying *sacB* used to construct gene deletion mutants; Ap^r (Tc^r)	(Li et al., 2015)

pHM1	2.2 kbp region upstream of C6N29_10910 amplified with primers 2245 and 2246 and inserted into KpnI and SalI sites of pMS75; Ap^r (Tc^r)	This study
pHM2	2.0 kbp region downstream of C6N29_10910 amplified with primers 2247 and 2248 and inserted into SalI and SphI sites of pHM1; Ap^r (Tc^r)	This study
pHM7	1.8 kbp region upstream of C6N29_08590 amplified with primers 2349 and 2350 and inserted into KpnI and SalI sites of pMS75; Ap^r (Tc^r)	This study
pHM8	2.0 kbp region downstream of C6N29_12020 amplified with primers 2353 and 2354 and inserted into BamHI and SphI sites of pMS75; Ap^r (Tc^r)	This study
pHM9	2.3 kbp region downstream of C6N29_00585 amplified with primers 2357 and 2358 and inserted into KpnI and SalI sites of pMS75; Ap^r (Tc^r)	This study
pHM10	2.3 kbp region downstream of C6N29_08590 amplified with primers 2351 and 2352 and inserted into SalI and SphI sites of pHM7; Ap^r (Tc^r)	This study
pHM11	2.1 kbp region upstream of C6N29_12020 amplified with primers 2355 and 2356 and inserted into SalI and SphI sites of pHM8; Ap^r (Tc^r)	This study
pHM12	2.7 kbp region upstream of C6N29_00585 amplified with primers 2359 and 2360 and inserted into SalI and SphI sites of pHM9; Ap^r (Tc^r)	This study
pHM13	2.3 kbp region downstream of C6N29_12115 amplified with primers 2445 and 2446 and inserted into KpnI and BamHI sites of pMS75; Ap^r (Tc^r)	This study
pHM15	1.8 kbp region downstream of C6N29_08680 (*tspA*) amplified with primers 2453 and 2454 and inserted into KpnI and BamHI sites of pMS75; Ap^r (Tc^r)	This study
pHM16	2.0 kbp region upstream of C6N29_06855 amplified with primers 2257A and 2258A and inserted into KpnI and BamHI sites of pMS75; Ap^r (Tc^r)	This study
pHM18	2.1 kbp region upstream of C6N29_12115 amplified with primers 2447 and 2448 and inserted into BamHI and PstI sites of pHM13; Ap^r (Tc^r)	This study
pHM19	2.1 kbp region upstream of C6N29_08680 (*tspA*) amplified with primers 2455 and 2456 and inserted into BamHI and PstI sites of pHM15; Ap^r (Tc^r)	This study
pHM20	2.4 kbp region downstream of C6N29_06855 amplified with primers 2459A and 2460A and inserted into BamHI and PstI sites of pHM16; Ap^r (Tc^r)	This study
pHM22	Plasmid for complementation of Δ*tspA*; 2.3 kbp region containing C6N29_08680 (*tspA*) amplified with primers 2498 and 2499 and inserted into SphI and KpnI sites of pCP23; Ap^r (Tc^r)	This study
pHM23	Plasmid for complementation of ΔC6N29_12115; 1.3 kbp region containing C6N29_12115 amplified with primers 2535 and 2536 and inserted into KpnI and SphI sites of pCP23; Ap^r (Tc^r)	This study

pNT21	2.1 kbp region downstream of C6N29_03390 amplified with primers 2262 and 2263 and inserted into KpnI and BamHI sites of pMS75; Ap^r (Tc^r)	This study
pNT22	2.1 kbp region upstream of C6N29_04605 amplified with primers 2266 and 2267 and inserted into KpnI and BamHI sites of pMS75; Ap^r (Tc^r)	This study
pNT23	2.2 kbp region upstream of C6N29_05315 amplified with primers 2270 and 2271 and inserted into KpnI and XbaI sites of pMS75; Ap^r (Tc^r)	This study
pNT24	2.1 kbp region downstream of C6N29_07780 amplified with primers 2274 and 2275 and inserted into KpnI and BamHI sites of pMS75; Ap^r (Tc^r)	This study
pNT25	2.1 kbp region downstream of C6N29_08145 amplified with primers 2278 and 2279 and inserted into KpnI and BamHI sites of pMS75; Ap^r (Tc^r)	This study
pNT26	2.1 kbp region downstream of C6N29_10335 amplified with primers 2286 and 2287 and inserted into KpnI and BamHI sites of pMS75; Ap^r (Tc^r)	This study
pNT28	2.1 kbp region downstream of C6N29_13645 amplified with primers 2298 and 2299 and inserted into KpnI and BamHI sites of pMS75; Ap^r (Tc^r)	This study
pNT30	2.5 kbp region upstream of C6N29_03390 amplified with primers 2264 and 2265 and inserted into BamHI and PstI sites of pNT22; Ap^r (Tc^r)	This study
pNT31	2.5 kbp region downstream of C6N29_04605 amplified with primers 2268 and 2269 and inserted into BamHI and PstI sites of pNT23; Ap^r (Tc^r)	This study
pNT32	2.5 kbp region downstream of C6N29_05315 amplified with primers 2272 and 2273 and inserted into XbaI and PstI sites of pNT23; Ap^r (Tc^r)	This study
pNT33	2.1 kbp region upstream of C6N29_08145 amplified with primers 2280 and 2281 and inserted into BamHI and PstI sites of pNT25; Ap^r (Tc^r)	This study
pNT36	2.6 kbp region upstream of C6N29_07780 amplified with primers 2276 and 2277 and inserted into BamHI and PstI sites of pNT24; Ap^r (Tc^r)	This study
pNT37	2.6 kbp region upstream of C6N29_10335 amplified with primers 2288 and 2289 and inserted into BamHI and PstI sites of pNT26; Ap^r (Tc^r)	This study
pNT39	2.5 kbp region downstream of C6N29_13645 amplified with primers 2300 and 2301 and inserted into BamHI and PstI sites of pNT28; Ap^r (Tc^r)	This study
pNT53	2.1 kbp region upstream of C6N29_09865 amplified with primers 2282 and 2283 and inserted into KpnI and BamHI sites of pMS75; Ap^r (Tc^r)	This study
pNT59	2.4 kbp region downstream of C6N29_09865 amplified with primers 2284 and 2285 and inserted into BamHI and PstI sites of pNT53; Ap^r (Tc^r)	This study
pNT76	Plasmid for chromosomal complementation of ΔC6N29_13645; 7.4 kbp fragment spanning C6N29_13645	This study

	and 2 kbp regions upstream and downstream amplified with primers 2298 and 2301 and inserted into KpnI and PstI sites of pMS75; Apr (Tcr)	
pRC27	2.1 kbp region upstream of C6N29_14605 amplified using primers 2330 and 2331 and inserted into KpnI and BamHI sites of pMS75; Apr (Tcr)	(Conrad, 2021)
pRC28	2.4 kbp region upstream of C6N29_03570 amplified using primers 2334 and 2335 and inserted into KpnI and BamHI sites of pMS75; Apr (Tcr)	(Conrad, 2021)
pRC31	2.1 kbp region downstream of C6N29_03570 amplified using primers 2336 and 2337 and inserted into BamHI and PstI sites of pRC28; Apr (Tcr)	(Conrad, 2021)
pRC34	2.4 kbp region downstream of C6N29_14605 amplified using primers 2332 and 2333 and inserted into BamHI and PstI sites of pRC27; Apr (Tcr)	(Conrad, 2021)

[a] Antibiotic resistance phenotypes: ampicillin, Ap^r; tetracycline, Tc^r. Unless indicated otherwise, the antibiotic resistance phenotypes are those expressed in *E. coli*. The antibiotic resistance phenotypes given in parentheses are those expressed in *F. columnare* but not in *E. coli*.

Table S3. Primers used to construct plasmids

Primer	Sequence (5' to 3')[a]	Plasmids constructed using this primer
2245	GCTAGGGATCCAGCATCTTTCTCAGGTATTG	pHM1
2246	GCTAGGTCGACTGAGGCAGTTGCACTTCTTGATCC	pHM1
2247	GCTAGGTCGACAATAACAACTTCCTCAGGGAA	pHM2
2248	GCTAGGCATGCGCGCTACCAATCTACCATCTATGA	pHM2
2262	GCTAGGGTACCCGTGTACATAACCTAAAACGGTTAGGG	pNT21
2263	GCTAGGGATCCGATATCACATCATTACATAACGGT	pNT21
2264	GCTAGGGATCCCTCATTGCTATTGGCTTTACCATG	pNT30
2265	GCTAGCTGCAGAGTTTATAAGAAATACCTTTGCGCG	pNT30
2266	GCTAGGGTACCTCGCAGATATAGTTTTGCTACATTAAGG	pNT22
2267	GCTAGGGATCCTGCTAATTGCTCTTTGATTAAATCGAC	pNT22
2268	GCTAGGGATCCGCTGGAAGCTACATATTAGAAGTT	pNT31
2269	GCTAGCTGCAGTATACATGCTGAGGGCTATCCAGC	pNT31
2270	GCTAGGGTACCAAGGTTCTGAACGAATTGAATCCG	pNT23
2271	GCTAGTCTAGAGGTCGAATTTACTCTATTACCATTAAC	pNT23
2272	GCTAGTCTAGAAGAACAGTTGATTTATCAGGATTA	pNT32
2273	GCTAGCTGCAGAGTAATAAGGCTTATGCCGAAATGC	pNT32
2274	GCTAGGGTACCGTTCGTTTAGTCCGCTTATGG	pNT24
2275	GCTAGGGATCCGATTTATCAAATTTAGCACCTGGA	pNT24
2276	GCTAGGGATCCGTCTCTTAGCATACCTACATCTCC	pNT36
2277	GCTAGCTGCAGAGTTGTATATCCGTGTCCTACTGC	pNT36
2278	GCTAGGGTACCGGGCATTTAGGATCAAGTTTAGGC	pNT25
2279	GCTAGGGATCCCGAATTGTTTATTCAGAAGAACTCAAC	pNT25
2280	GCTAGGGATCCGATGAGGTCAATATTGCTTTGACTAAG	pNT33
2281	GCTAGCTGCAGGACTATCAATACACCGAAGCCAGC	pNT33
2282	GCTAGGGTACCGCTAAATCCGTGGCTGTACCTATG	pNT53
2283	GCTAGGGATCCTTCCTGCATATAGGATTCCGTACC	pNT53
2284	GCTAGGGATCCGCTATTCAAATTGTAGATCGCATAGGG	pNT59
2285	GCTAGCTGCAGGTACAGAAGGCAACTGGCTTC	pNT59
2286	GCTAGGGTACCTACTCCTGTCTTGTTTAATGCC	pNT26
2287	GCTAGGGATCCACCCTTAAATTCAACAATGAAGGT	pNT26
2288	GCTAGGGATCCTTCCGCTTCATATTCAACAGTTGA	pNT37
2289	GCTAGCTGCAGCTCTGTTGTAGTGTAAGTAGCTCC	pNT37
2298	GCTAGGGTACCATCGTACCATTTCGGCTTCAC	pNT28, pNT76
2299	GCTAGGGATCCGGTTCTGTCTTTAATAATGAGATAGAT	pNT28
2300	GCTAGGGATCCTTCTTGTGCTTGCAGATTTGAAAT	pNT39
2301	GCTAGCTGCAGGCTGTATAAATTCCTCCCACAG	pNT39, pNT76

2330	GCTAGGGTACCTCTGTATAAGGCCTACGGC	pRC27
2331	GCTAGGGATCCTCCTCCTGTAACAGTTTCCTC	pRC27
2332	GCTAGGGATCCGTAGGAGGAAGGGATAGAATTCAT	pRC34
2333	GCTAGCTGCAGATGAAGCAGTCCATTGATCG	pRC34
2334	GCTAGGGTACCGCCTCTCCTAGAACTTCTATTTCA	pRC28
2335	GCTAGGGATCCGGGAATCCACATTCCGCC	pRC28
2336	GCTAGGGATCCATCGTCCAACCAATAGGAACT	pRC31
2337	GCTAGCTGCAGGCAAAACGAAACTATTGCAGG	pRC31
2349	GCTAGGGTACCAATGATACTAGTACCGATGCC	pHM7
2350	GCTAGGTCGACAGATAATTGAGCTGCATCCTCATC	pHM7
2351	GCTAGGTCGACAGAGCAGAATCAGAAGGGATC	pHM10
2352	GCTAGGCATGCAGGATTGATACTGGTGCTGTCTATATC	pHM10
2353	GCTAGGGATCCTCTCAAGAAGATAACTCAACAACC	pHM8
2354	GCTAGGTCGACATGAACTATATGGATTACACTGACGAC	pHM8
2355	GCTAGGTCGACTGCACATCCTCTATGAGCAGC	pHM11
2356	GCTAGGCATGCATCATCCAATCCGCCATG	pHM11
2357	GCTAGGGTACCTGTAGCAGGAATGAATGTAGACATATC	pHM9
2358	GCTAGGTCGACGATGACAGAGTAGTACCAGCACAC	pHM9
2359	GCTAGGTCGACTCCAAAATAGGTATCAACATGTTC	pHM12
2360	GCTAGGCATGCCATAAGTATGATCATTCGCATACG	pHM12
2445	GCTAGGGTACCGTCTACCAGGAAACTTAATTCG	pHM13
2446	GCTAGGGATCCGACTACACAGATGACAGAGGAATG	pHM13
2447	GCTAGGGATCCGTGAGAAGCACATCCTCTGTG	pHM18
2448	GCTAGCTGCAGTCGTAAATACGCATCTATTAGTCGTGC	pHM18
2453	GCTAGGGTACCTCCTCCATATTGTTCAGAACTCAATCC	pHM15
2454	GCTAGGGATCCTGGTTTGAAGCTATTACAAAAGATGCC	pHM15
2455	GCTAGGGATCCTACAATAGCGTCTAGTAGAACTCGATC	pHM19
2456	GCTAGCTGCAGTTCTAAAGTAGCCGTTCCTGAAGTTAC	pHM19
2457A	GCTAGGGTACCACTTAATGCGAAACTCCAACC	pHM16
2458A	GCTAGGGATCCAGCACTATCAGCACTTACTGAAGG	pHM16
2459A	GCTAGGGATCCATTCATTCACCTAATGAACATTATGGT	pHM20
2460A	GCTAGCTGCAGCGTTATCAATTCTATGAGGACAACACG	pHM20
2498	GCTAGGCATGCGCTTCTTCGTGTTGTTACATC	pHM22
2499	GCTAGGGTACCATTATACCATCAATTTTATATTCGCAT	pHM22
2535	GCTAGGGTACCAACAAGAAACCTTTGCTTTGTTC	pHM23
2536	GCTAGGCATGCTCCTGAGAAACAACCTCAGGAT	pHM23

[a] Underlined sequences indicate introduced restriction enzyme sites

References:

Agarwal, S., Hunnicutt, D.W., and McBride, M.J. (1997). Cloning and characterization of the *Flavobacterium johnsoniae* (*Cytophaga johnsonae*) gliding motility gene, *gldA*. *Proc. Natl. Acad. Sci. USA* 94**,** 12139-12144.

Bartelme, R.P., Barbier, P., Lipscomb, R.S., LaPatra, S.E., Newton, R.J., Evenhuis, J.P., et al. (2018). Draft genome sequence of the fish pathogen *Flavobacterium columnare* strain MS-FC-4. *Genome Announc* 6(20)**,** e00429-00418. doi: 10.1128/genomeA.00429-18.

Conrad, R.A. (2021). *Roles of secreted proteins and iron utilization proteins in virulence of the fish pathogen Flavobacterium columnare.* Ph. D., University of WI-Milwaukee.

de Lorenzo, V., and Timmis, K.N. (1994). Analysis and construction of stable phenotypes in gram-negative bacteria with Tn5- and Tn10-derived minitransposons. *Methods Enzymol* 235**,** 386-405.

Evenhuis, J.P., and LaFrentz, B.R. (2016). Virulence of *Flavobacterium columnare* genomovars in rainbow trout *Oncorhynchus mykiss*. *Dis Aquat Organ* 120(3)**,** 217-224. doi: 10.3354/dao03027.

Kaplan, E.L., and Meier, P. (1958). Nonparametric estimation from incomplete observations. *J. Amer. Statist. Assoc.* 53**,** 457-481. doi: 10.2307/2281868.

Li, N., Qin, T., Zhang, X.L., Huang, B., Liu, Z.X., Xie, H.X., et al. (2015). Gene deletion strategy to examine the involvement of the two chondroitin lyases in *Flavobacterium columnare* virulence. *Appl Environ Microbiol* 81(21)**,** 7394-7402. doi: 10.1128/AEM.01586-15.

Thunes, N.C., Conrad, R.A., Mohammed, H.H., Zhu, Y., Barbier, P., Evenhuis, J.P., et al. (2022). Type IX secretion system effectors and virulence of the model *Flavobacterium columnare* strain MS-FC-4. *Appl Environ Microbiol* 88(3)**,** e0170521. doi: 10.1128/AEM.01705-21.
